# Intestinal carbapenem-resistant *Klebsiella pneumoniae* undergoes complex transcriptional reprogramming following immune activation

**DOI:** 10.1080/19490976.2024.2340486

**Published:** 2024-04-24

**Authors:** Clement David, Aleksander Czauderna, Liqing Cheng, Marion Lagune, Hea-Jin Jung, Sohn G. Kim, Eric G. Pamer, Julien Prados, Liang Chen, Simone Becattini

**Affiliations:** a Department of Pathology and Immunology, Faculty of Medicine, University of Geneva, Geneva, Switzerland; b Geneva Centre for Inflammation Research, Faculty of Medicine, University of Geneva, Geneva, Switzerland; c Immunology Program, Sloan Kettering Institute, Memorial Sloan-Kettering Cancer Center, New York, NY, USA; d Duchossois Family Institute, University of Chicago, Chicago, IL, USA; e Bioinformatics Support Platform for data analysis, Faculty of medicine, University of Geneva, Geneva, Switzerland; f Department of Pharmacy Practice, School of Pharmacy and Pharmaceutical Sciences, University at Buffalo, Buffalo, NY, USA

**Keywords:** Carbapenem-resistant *Klebsiella pneumoniae*, transcriptomics, gut, immunity

## Abstract

Carbapenem-resistant *Klebsiella pneumoniae* (CR-Kp) is a significant threat to public health worldwide. The primary reservoir for CR-Kp is the intestinal tract. There, the bacterium is usually present at low density but can bloom following antibiotic treatment, mostly in hospital settings. The impact of disturbances in the intestinal environment on the fitness, survival, expansion, and drug susceptibility of this pathogen is not well-understood, yet it may be relevant to devise strategies to tackle CR-Kp colonization and infection. Here, we adopted an *in vivo* model to examine the transcriptional adaptation of a CR-Kp clinical isolate to immune activation in the intestine. We report that as early as 6 hours following host treatment with anti-CD3 antibody, CR-Kp underwent rapid transcriptional changes including downregulation of genes involved in sugar utilization and amino acid biosynthesis and upregulation of genes involved in amino acid uptake and catabolism, antibiotic resistance, and stress response. In agreement with these findings, treatment increased the concentration of oxidative species and amino acids in the mouse intestine. Genes encoding for proteins containing the domain of unknown function (DUF) 1471 were strongly upregulated, however their deletion did not impair CR-Kp fitness i*n vivo* upon immune activation. Transcription factor enrichment analysis identified the global regulator cAMP-Receptor Protein, CRP, as a potential orchestrator of the observed transcriptional signature. In keeping with the recognized role of CRP in regulating utilization of alternative carbon sources, *crp* deletion in CR-Kp resulted in strongly impaired gut colonization, although this effect was not amplified by immune activation. Thus, following intestinal colonization, which occurs in a CRP-dependent manner, CR-Kp can rapidly respond to immune cues by implementing a well-defined and complex transcriptional program whose direct relevance toward bacterial fitness warrants further investigation. Additional analyses utilizing this model may identify key factors to tackle CR-Kp colonization of the intestine.

## Introduction

*Klebsiella pneumoniae* (Kp) is a gram-negative, non-motile rod, endowed with a prominent sugar capsule, which causes human respiratory, urinary tract, and systemic infections.^1^ Currently, there are no vaccines available against Kp, and antibiotic therapy remains the standard intervention strategy in case of infection. However, due to the large number of antibiotic resistance genes (ARGs) acquired by Kp strains over the last decade, treatments have become largely ineffective. In fact, Kp is currently listed amongst the most dangerous nosocomial multidrug-resistant (MDR) pathogens, grouped under the acronym ‘ESKAPE’,^2^ and considered a global health priority.^3^ In particular, a single Kp strain known as ST (Sequence Type) 258 has acquired resistance to carbapenems, last-resort antibiotics for the treatment of MDR infections, and has spread globally, becoming endemic in multiple countries.^1,3–5^

Carbapenem resistance is primarily achieved through the production of carbapenemases, such as the *Klebsiella pneumoniae* carbapenemase (KPC), the New Delhi metallo-beta-lactamase (NDM), and the OXA-48-like enzymes.^1^

The gastrointestinal (GI) tract is a major reservoir of Kp, including carbapenem-resistant (CR)-Kp strains, and overgrowth promoted by antibiotic treatment, can favor infection both in humans and animal models and spread amongst hospitalized patients.^6–10^ Disseminated infections caused by CR-Kp are extremely difficult to treat due to antibiotic inefficacy.^8,11^ Understanding the molecular mechanisms that regulate CR-Kp fitness in the gut lumen is therefore of primary importance, and multiple animal models have been established to investigate this issue.^6,8,12–15^ Several variables can affect fitness and propagation of pathobionts in the intestinal microenvironment. For instance, commensal microbes can directly or indirectly limit the capacity of Kp to colonize the intestinal lumen.^6,14,16^ In addition, host immunity is a key modulator of *Enterobacteriaceae* fitness in the gut.^17^ Landmark studies have uncovered that, by producing reactive oxygen and nitrogen species, inflammation can boost the luminal expansion of bacterial species capable of using those substrates for respiration, such as *E. coli* and Salmonella spp.^18,19^ Similarly, the capacity to metabolize amino acids such as L-serine, whose luminal concentrations increase in the inflamed intestine, favors *E. coli* outgrowth in mouse models.^20^ In line with these notions, *Klebsiella* species are significantly more abundant in the intestine of patients with inflammatory bowel disease (IBD) and can even promote colitis in a variety of animal models.^21–27^

Despite the clinical relevance of CR-Kp in the context of intestinal colonization and inflammation, the molecular programs adopted by this pathobiont in the gut lumen, and their relations with the immune status of the host, have not been investigated. Unraveling the mechanisms through which CR-Kp adapts to inflammatory cues in the intestine may suggest tailored approaches to facilitate clearance of this relevant pathobiont.

Here, we performed transcriptional profiling of intestinal CR-Kp ST-258 (MH-258) in mice undergoing acute immune activation and identified bacterial genes that were subsequently differentially regulated. The immunity-induced transcriptional program of CR-Kp is established rapidly and encompasses switches in carbon source utilization, activation of stress response mechanisms, and modulation of antibiotic resistance. Importantly, a core transcriptional signature was confirmed through experiments carried out in distinct *vivaria*, implying its robustness. We report that genes encoding for protein containing a domain of unknown function common to multiple paralogs within the *Enterobacteriaceae* family (DUF1471) are strongly induced by host immunity. Selective deletion of these genes did not reduce the capacity of CR-Kp to colonize the inflamed intestine, suggesting that they may serve other, unknown functions. On the contrary, we describe that the cAMP-Receptor Protein (CRP), a predicted master regulator of the above transcriptional signature, is strictly required to establish intestinal colonization in the first place.

Our work sets the stage for a comprehensive understanding of functional regulation in intestinal CR-Kp on the basis of host immune status. This knowledge may assist the development of novel therapeutic approaches to tackle CR-Kp intestinal carriage and bloom via rational modulation of the gut microenvironment.

## Methods

### Genome, plasmids, primers, spacers, donor DNA, Kp bacterial strains, and growth conditions

The genome of the *Klebsiella* strain utilized in this study was annotated using PATRIC, which employs the RAST tool kit.^28^ NCBI gene identifiers for RAST-predicted peg (protein encoding genes) are provided in Supplementary Table S2.

All the plasmids, bacterial strains, and cell lines used in this study are listed in Supplementary Table S3. All the primers used in this study are listed in Supplementary Table S4. The spacers and DNA donors used in this study are listed in Supplementary Table S5. The primers used in this study were purchased from Microsynth (Balgach, Switzerland) and from LubioScience GmbH (Zürich, Switzerland). *K. pneumoniae* strains were grown in lysogeny broth (LB) medium (per liter, 5 g of yeast extract, 10 g of tryptone, 10 g of NaCl [pH 7.2 to 7.4]). Antibiotics were added to the following concentrations: 30ug/ml apramycin, 100ug/ml rifampicin, 50 ug/ml neomycin. Unless otherwise noted, we utilized bacteria in the early log phase (OD = 0.1–0.4) from sub-inoculations of overnight cultures. A linear relation between OD values and CFUs is maintained in this range.

The temperature-sensitive pCasKp plasmid and the pSGKp-Rif were provided by Dr. Liang Chen (Hackensack Meridian School of Medicine & Center for Discovery and Innovation) (Wang, Y. et al., 2018).

### Mouse husbandry

All experiments using wild-type mice were performed with C57BL/6J female mice that were 6–9 weeks old; mice were purchased from Charles Rivers. All mice were housed in a sterile, autoclaved cages with irradiated food and autoclaved water. Mouse handling and cage changes were performed by investigators wearing gloves, masks, and sterile gowns. All mice were maintained in a specific-pathogen-free facility at the Centre Médical Universitaire (CMU) of the University of Geneva, Switzerland.

All of the animal procedures and experiments were approved and performed in accordance with the guidelines of the animal research committee of Geneva, Switzerland.

### Mouse intestinal reconstitution and intranasal inoculation

Kp strains (i.e. WT ST258 and derived mutants) were maintained in glycerol stock at −80°C. To generate the inoculum to be administered to mice, bacteria were inoculated in LB-broth supplemented with carbenicillin (100µg/ml) and neomycin (50ug/ml) and incubated overnight at 37°C 220rpm. O.n. cultures were sub-inoculated 1:500 in fresh LB medium and incubated at 37°C 220rpm. At OD ~0.1–0.4, bacteria were washed once with ice-cold PBS.

For mouse reconstitution, bacteria were resuspended in ice-cold PBS, to prevent further replication, at a concentration of 0.5 × 10^6^CFU/ml. About 200µl of bacterial mixture (0.1×10^5^CFUs) was administered to ampicillin-treated mice by oral gavage.

Depending on the local microbiota flora, mice were administered either ampicillin only (0.5 g/l, MSKCC facility) or AMNV in drinking water (ampicillin, metronidazole, neomycin, vancomycin; 0.5 g/l each, CMU facility) starting 3 days prior to reconstitution with Kp. After oral inoculation of Kp, AMNV in drinking water was replaced with ampicillin (0.5 g/l) for the whole duration of the experiment.

### Preparation of competent cells and electroporation

The preparation of Kp competent cells and electroporation of Kp were performed as described previously (Wang, Y. et al., 2018). Briefly, 1 ml of Kp WT overnight culture was diluted into 100 ml of LB broth and incubated at 37°C. When the optical density at 600 nm (OD600) of the cell culture reached ~ 0.5–0.7, the culture was chilled on ice for 20 min and harvested by centrifugation at 7,200 × g for 5 min. The supernatant was discarded, and the cells were resuspended with 5 ml of sterile ice-cold 10% glycerol. The centrifugation and resuspension steps were repeated twice. Finally, the cells were resuspended with 1 ml of ice-cold 10% glycerol. Fifty-microliter aliquots were frozen and stored at −80°C.

For the pCasKP-harboring *K. pneumoniae* strain, 1 ml of overnight culture from a fresh single colony was diluted into 100 ml of LB broth containing 30ug/ml apramycin and incubated at 30°C. When the cell density reached an OD600 of ~0.2, 1 ml of 20% L-arabinose was added for induction of the lambda Red recombineering operon of pCasKP. After induction at 30°C for 2 h, the culture was prepared as electrocompetent cells in a way similar to that of the WT Kp.

For electroporation, 50 µl of electrocompetent cells were thawed on ice for several minutes. Then, the cells were mixed with no more than 5 µl plasmid or donor template. The mixture was transferred into a 2-mm electroporation cuvette (BTX) and electroporated at 2.5 kV, 200Ω, and 25uF using an ECM 630 electroporation system (BTX). After being pulsed, the cells were recovered in 1 ml antibiotic-free LB broth and incubated at 30°C for 1.5 h before being plated onto LB agar plates supplemented with the required antibiotics. The plates were incubated at 30°C overnight.

### Genome editing

The pSGKp plasmid was extracted from *Escherichia coli* DH5α using the PureLink™ Quick Plasmid Miniprep Kits (Invitrogen). Plasmid was linearized with BsaI (New England Biolab) into FastDigest Buffer (10X) (ThermoFisher) for 20 min at 37°C. Then the plasmid was gel purified using the GeneJET PCR purification kit (Thermoscientific) and quantified by nanodrop. In parallel, phosphorylation and annealing of the spacers were performed as follows: 5 µl of spacer forward and 5µl spacer reverse were incubated for 30 min at 37°C with 5 µl 10× T4DNA Ligase (NEB) and 1µl T4 polynucleotide kinase (NEB) in a 50 µl reaction. The spacers were then ligated for 1 h into the linearized pSGKp plasmid through a 10 µl reaction at room temperature, in presence of 1µl of 10× T4DNA Ligase Buffer (NEB) and 1µl of T4 DNA Ligase (NEB). The ligated product was then transformed in *E. coli* DH5α ECO112 (ThermoScientific) following manufacturer’s instructions. The bacteria were grown overnight at 37°C on LB-agar supplemented with 100ug/ml Rifampicin. Transformed colonies were identified through colony PCR using DreamTaq Green PCR (ThermoScientific) and confirmed by Sanger sequencing (Microsynth). Transformed clones were stored in glycerol stock at −80°C.

When needed, pSGKp containing the spacers was extracted from *E. Coli* DH5α overnight cultures using the PureLink™ Quick Plasmid Miniprep Kits (Invitrogen). The extracted plasmid was used for electroporation. After electroporation and overnight culture of electroporated Kp, the mutagenesis was checked through colony PCR and Sanger sequencing as explained before. Then, either only the pSGKp plasmid was cured from Kp or both pCasKp and pSGKp were cured.

For the curing of the pSGKp, bacteria were grown at 30°C overnight in LB broth supplemented with 30ug/ml apramycin and 5% sucrose. Then, bacteria were grown at 30°C overnight on LB-agar supplemented with 30ug/ml apramycin (to check the presence of the pCasKp plasmid) and LB-agar supplemented with 30ug/ml apramycin and 100ug/ml rifampicin (to check the absence of the pSGKp plasmid). The mutant bacteria that had successfully cured the pSGKp plasmid were used for another round of electroporation.

For the curing of both plasmid, bacteria were grown at 37°C overnight in LB broth. Then, they were plated on LB-agar supplemented with 5% sucrose. Finally, clones were picked and streaked on LB-agar plates supplemented with 30 µg/ml apramycin, LB-agar plates supplemented with 100 µg/ml with rifampicin and LB-agar plates (to confirm successful curing).

### Immune stimulation

Kp-reconstituted WT mice were injected i.p. with anti-CD3 antibody (clone 145-2C11, BioXCell; 100ug/mouse in 200 µl PBS) and sacrificed at the indicated time points.

### Dextran sodium sulfate immune stimulation

Kp-reconstituted WT mice were administered Dextran Sodium Sulfate (DSS) ad-libitum in water (3% DSS in water) and sacrificed at the indicated time points. Fecal pellets were sampled at the indicated time points and were mechanically homogenized (bead beating). Seven serials 1:10 dilutions of the mixture were plated onto LB-agar supplemented with carbenicillin (100ug/ml) and neomycin (50ug/ml). After 24 h incubation at 37°C, CFUs were counted.

### Lung infection and organ processing

For intranasal inoculation, bacteria were resuspended in ice-cold PBS at a concentration of 1 × 10^8^CFU/ml. 40 µl of bacteria mixture was administered intranasally to mice anesthetized with isoflurane. Intranasally inoculated mice were sacrificed 24 h after intranasal inoculation. Lungs were mechanically homogenized (bead beating) and 8 serials 1:10 dilutions of the mixture were spot-plated onto LB-agar supplemented with carbenicillin (100 µg/ml) and neomycin (50 µg/ml).

### RNA extraction (mouse tissue, murine cell lines)

RNA was isolated from cecum tissue or cell lines, using mechanical homogenization (bead beating) and TRIzol isolation. Briefly, samples were placed into 1 ml of TRIzol (Invitrogen), bead homogenized, and spinned down for 10 min at 12’000 × g 4°C. Then, 0.2 ml of chloroform were added to the tube, followed by 15 sec of vortexing, and incubation at room temperature for 2-3 min. Tubes were centrifuged for 15 min at 12’000 × g 4°C. The RNA was then precipitated transferring the aqueous phase into a new tube and adding 0.5 ml isopropanol. The mixture was incubated at room temperature for 10 min, followed by a centrifugation at 12’000 × g 4°C for 10 min. Finally, the RNA was washed by removing the supernatant and adding 1 ml of 75% ethanol. RNA was pelleted at 7’500 × g 4°C for 5 min, supernatant was removed, and the RNA pellet was air dried for 5 min at room temperature. Then the RNA was resuspended in 100 µl of nuclease-free water and stored at −80°C.

### RNA extraction (Kp RNA)

Cecal content was extracted from dissected mice immediately after sacrificed and snap-frozen in dry ice. RNA was isolated from fecal pellets or cecal contents using the RNeasy PowerMicrobiome Kit (Qiagen), following the manufacturer’s instructions. RNA was eluted in 100 µl nuclease-free water and stored at −80°C.

### RT qPCR (mouse)

RNA was isolated from cecum tissue or RAW 267.4 macrophages, using mechanical homogenization and TRIzol isolation, as described above. cDNA was obtained using the iScript Reverse Transcription Supermix (Bio-Rad). RT-qPCR was performed on cDNA using the PowerUP SYBR Green Master Mix (Applied Biosystems) and custom primers (Supplementary Table S3). Gene expression was normalized to expression of GAPDH in the control group. Reactions were run on a QuantStudio 6 PRO (Applied Biosystems). The cycling conditions were as follows: 95°C for 20 sec, followed by 40 cycles of 95°C for 1 sec and 60°C for 20 sec. A melt curve has been performed after the 40 cycles as follows: 95°C for 1 sec, 60°C for 20 sec and 95°C for 1 sec.

### RT qPCR (Klebsiella)

Kp RNA was extracted from fecal pellets or cecal content as indicated above, cDNA was generated using the QuantiTect reverse transcriptase kit (Qiagen). qPCR was carried out using the PowerUP SYBR Green Master Mix (Applied Biosystems) and dedicated custom primers for the target genes and housekeeping genes selected from our RNA-seq datasets based on low variance in expression despite host immune activation (Supplementary Table S3). Reactions were run on a QuantStudio 6 PRO (Applied Biosystems). The cycling conditions were as follows: 95°C for 20 sec, followed by 40 cycles of 95°C for 1 sec and 60°C for 20 sec. A melt curve was generated after the 40 cycles using the following conditions: 95°C for 1 sec, 60°C for 20 sec and 95°C for 1 sec.

### DNA extraction

Kp DNA was extracted from fecal pellets or cecal contents or overnight liquid cultures (12 h LB broth supplemented with carbenicillin (100ug/ml) and neomycin (50ug/ml)) by mechanical disruption (bead beating), followed by extraction using the QIAamp PowerFecal Pro DNA Kits (Qiagen), according to manufacturer’s instructions. After extraction, DNA was eluted in 100 µl nuclease-free water and store at −20°C.

### qPCR to measure competitive index

Kp DNA extracted from fecal pellets or cecal content was subjected to quantitative PCR using the following custom Kp member-specific primers (Supplementary Table S3).

868 WT: fwd: 5’-tttagccgccagttcctgac-3’; rev: 5’-ttctctctttcggtgccagc-3’; 868KO: fwd: 5’-cgacgagggtaatctttact-3’; rev: 5’-ttcgacgaggtgaacatcgt-3’ (for WT vs 6KO competition).

CRPWT: fwd: 5’-ggttgcccactttctcagac-3’; rev: 5’-cctgtttgaagagggtcagg-3’; CRPKO: fwd: 5’-acgccgcttagcaacgcgcggtt-3’; rev: 5’-tgctaaaacagtctggatgct-3’ (for WT vs CRPKO competition).

Standard curves were generated by amplification of DNA extracted from pure overnight liquid culture (12 h LB-broth supplemented with carbenicillin (100ug/ml) and neomycin (50ug/ml)) of the WT Kp, the 6KO Kp and the Δcrp Kp (six serials 1:10 dilutions, from 3.3ng/ µl, 3 µl, ~10ug total DNA). Internal controls consisting of equal amount of WT DNA and mutant DNA (50:50, WT:6KO or WT: Δcrp), and different ratios od WT DNA over mutant DNA (75:25, WT:6KO or WT: Δcrp; 25:75, WT:6KO or WT: Δcrp), were always performed to confirm accuracy of the qPCR. PowerUP SYBR Green Master Mix (Applied Biosystems) was used to perform the reactions. Reactions were run on a QuantStudio 6 PRO (Applied Biosystems). The cycling conditions were as follows: 95°C for 20 sec, followed by 40 cycles of 95°C for 1 sec and 60°C for 20 sec. A melt curve was generated after the 40 cycles as follows: 95°C for 1 sec, 60°C for 20 sec, and 95°C for 1 sec.

### RNA sequencing and analysis (Kp RNA)

Kp RNA sample libraries were prepared using the Illumina Stranded Total RNA Prep kit with the Ribo-zero Plus and sequenced using the Illumina NovaSeq 6000 with single-end reads 100bp (experiment CMU1), paired-end reads 100 bp (MSK1) or MiSeq with paired-end reads 150 bp (experiment MSK2). Raw sequence reads were filtered using Trimmomatic (v.0.39),^29^ sequentially aligned to the mouse genome to remove mouse-derived sequences and then to the Kp genome using bowtie2 (v.2.2.5).^30^ BAM files obtained were analyzed using R (v.4.0.3). Reads were assigned to genes using FeatureCounts from the Rsubread package (v.2.4.2).^31^ Reads were then analyzed using ‘DeSeq2’ package (v.1.30.0).^32^ GSEA was performed using the package ‘clusterProfiler’ (v.4.6.0).^33^

### RNA sequencing and analysis (murine RNA)

Murine RNA sample libraries for experiments CMU1 were prepared using the Illumina Stranded Total RNA Prep kit with the Ribo-zero Plus and sequenced using the Illumina NovaSeq 6000 with single read 100bp. Libraries were prepared on a 1:6 mixture of RNA extracted from the mouse tissue and from the respective bacteria, so that each library would produce both mouse and *Klebsiella*-derived reads. Raw sequence reads were aligned to the reference mouse genome UCSC mm10 using STAR (v.2.7.4.a). Reads were assigned to genes using HTSeq counts (v.0.9.1). Reads were then analyzed in R (v.4.0.3) using ‘DeSeq2’ package (v.1.30.0). GSEA was performed using the package ‘clusterProfiler’ (v.4.6.0) using R (v.4.2.2).

### Transcription factors enrichment analysis

A generic database of 201 transcription factors (TF) motifs of prokaryote genomes was downloaded from MEME suite website (https://meme-suite.org/meme/db/motifs, Prodoric database). The DNA sequence of our *Klebsiella pneumoniae* genome was scanned with these motifs using function scan_sequences() from R Bioconductor package universalmotif. All hits matching the genome with a p-value lower than 1e-6 were collected (*n* = 71459 hits), and we associate to each TF, the genes that have a hit for its motif in their promoter region (defined as 0-500bp upstream of the gene). This process builds our regulon database on which we conducted enrichment analysis tests, with a hypergeometric test to assert whether differentially expressed genes found by RNA-seq were enriched for some TF.

### H_2_O_2_ assays

Kp strains were grown overnight in LB broth supplemented with carbenicillin (100 ug/ml) and neomycin (50 ug/ml) at 37°C, and then diluted 1:200 in fresh LB broth supplemented with the very same antibiotics at 37°C. At O.D. ~0.1–0.4, cells were diluted 1:100 in LB broth and challenged with 2.5 mM H2O2 for 1 h at 37°C. Viability of Kp strains was assessed through plating of serial 1:10 dilutions on LB-agar plates.

Stimulations for qPCR were carried out as follows. Kp strains were grown overnight in LB broth supplemented with carbenicillin (100 ug/ml) and neomycin (50 ug/ml) at 37°C and then diluted 1:200 in fresh LB broth supplemented with the very same antibiotics at 37°C. At O.D. ~0.6, 1 ml of Kp culture was mixed with 1 ml of LB-broth supplemented with 2.5 mM (H_2_O_2_) (final H_2_O_2_ concentration 1.25 mM) and incubated for 15 min at 37°C. Bacteria were spinned down at 3000 rpm for 10 min at 4°C, the supernatant was removed, and the cells were resuspended into TRIzol before proceeding with a RNA extraction as indicated above.

### H_2_O_2_ measurement in cecal content

WT mice were treated with AMNV for 4 days and then stimulated with anti-CD3 antibody (100ug/200 µl PBS) i.p. injection. 5 h post-injection, mice were sacrificed and cecal contents were collected and resuspended in PBS pH 7.4. Cecal contents were centrifuged at 3’000rpm for 10 min at 4°C. 40 µl of supernatant was mixed with 10 µl of 5 × concentrated mix of AmplexRed (0.125 mM) and horseradish peroxidase (0.5 U/ml). The mixture was incubated for 30 min at RT in the dark. Concentration of H_2_O_2_ was then determined by measuring the fluorescence at 590 nm on a SpectraMax Paradigm MultiMode Microplate Reader (Molecular Devices).

### Serum killing assay

Kp strains were grown overnight in LB broth supplemented with carbenicillin (100 µg/ml) and neomycin (50ug/ml) at 37°C and then diluted 1:200 in fresh LB broth supplemented with the very same antibiotics at 37°C. At O.D. ~0.1–0.4, cells are diluted 1:100 in LB broth and challenged with activated human serum for 1 h at 37°C. Viability of Kp strains was assessed through plating of serial 1:10 dilutions on LB-agar plates.

### Bile acids stress assay

Kp strains were grown overnight in LB broth supplemented with carbenicillin (100 µg/ml) and neomycin (50 µg/ml) at 37°C, and then diluted 1:200 in fresh LB broth supplemented with the very same antibiotics at 37°C. At O.D. ~0.1–0.4, viability to bile acids of Kp strains was assessed through plating of serial 1:10 dilutions on LB-agar plates supplemented with different concentration of bile acids (Cholic acid: 0.05%, 0.1%; Deoxycholic acid: 0.05%, 0.1%, 0.2%). After 24 h incubation at 37°C, CFUs were counted.

### Macrophage killing assay

RAW264.7 macrophages were grown in DMEM high glucose + 10% FBS + 1% Pen/Strep. At 90–100% confluence, RAW264.7 cells were seeded at 10^6^/ml in a 96-well plate and incubated overnight at 37°C. For the Interferon-γ (IFN- γ) stimulation: the culture medium was supplemented with 100 ng/ml IFN- γ for the whole experiment. In parallel, Kp strains were grown overnight at 37°C 220rpm, in LB broth supplemented with carbenicillin (100 µg/ml) and neomycin (50ug/ml). Then, Kp cultures were diluted 1:200 in fresh LB medium. At OD600 ~ 0.1–0.4, bacteria were washed twice with PBS by centrifuging at 3’000rpm 4°C, followed by a resuspension in antibiotic-free complete DMEM. Macrophages were washed once with PBS and antibiotic-free DMEM + 10% FBS was added. Bacteria were added to wells at MOI = 10 in 30 µl, and spin at 200 × g 5 min. Macrophages were incubated with Kp strains for 1 h at 37°C and wash twice with PBS. Then, cells were incubated with DMEM + 10% FBS supplemented with 300 ug/ml gentamycin for 30 min 37°C, to kill extracellular bacteria. Following incubation, cells were washed again twice with PBS and incubated for 3 h at 37°C in DMEM + 10% FBS supplemented with 50 ug/ml gentamycin. To assess recovery of intracellular bacteria, cells were washed twice with PBS and lysed with PBS + 0.1% Triton X-100, then the mixture was serial 1:10 diluted seven times and plated onto LB-agar plates. After 24 h incubation at 37°C, CFUs were counted.

### Macrophage activation assay

RAW264.7 macrophages were grown in DMEM high glucose + 10% FBS + 1% Penicillin/Streptomycin (Pen/Strep) (ThermoFisher). At 90–100% confluence, RAW264.7 cells were seeded at 0.5 × 10^6^/ml in a 48 well-plate and incubated overnight at 37°C. In parallel, Kp strains were grown overnight at 37°C 220rpm, in LB broth supplemented with carbenicillin (100ug/ml) and neomycin (50ug/ml). Then, Kp cultures were diluted 1:200 in fresh LB medium. At OD600 ~0.1–0.4, bacteria were washed twice with PBS by centrifuging at 3’000rpm 4°C, followed by a resuspension in antibiotic-free complete DMEM. Macrophages were washed once with PBS and antibiotic-free DMEM + 10% FBS was added. Bacteria were added to wells at MOI = 10 in 30ul and spun at 200 × g 5 min. Macrophages were incubated with Kp strains for 1 h at 37°C and washed twice with PBS. Then cells were incubated with DMEM + 10% FBS supplemented with 100 µg/ml gentamycin for 30 min 37°C, to kill extracellular bacteria. Following incubation, cells were washed again twice with PBS and incubated for 3 h at 37°C in DMEM + 10% FBS supplemented with 50ug/ml gentamycin. To assess the gene expression in macrophages, RNA was extracted with TRIzol and RT-qPCR was performed on the target genes, as explained previously.

### Antibiotic susceptibility assay

Kp strains were grown overnight in LB broth supplemented with carbenicillin (100 µg/ml) and neomycin (50 µg/ml) at 37°C, and then diluted 1:200 in fresh LB broth supplemented with the very same antibiotics at 37°C. At O.D. ~0.8–1, cells were diluted 1:1000 in LB broth and challenged for 3 h at 37°C with the following antibiotics: meropenem (100 µg/ml) and colistin (64ug/ml). Viability of Kp strains was assessed through plating of serial 1:10 dilutions on LB-agar plates.

### Growth curve in vitro

Kp strains were grown overnight in M9 medium supplemented with 0.5% D-glucose, carbenicillin (100 µg/ml) and neomycin (50ug/ml) at 37°C, and then diluted 1:250 in fresh M9 medium. 100 µl of this dilution were then put ontop of 100 µl M9 medium supplemented with different carbon sources (i.e. 2% D-glucose or 2% D-mannose or 2% L-serine+L-threonine+L-leucine) or ontop of 100 µl LB broth, in a flat-bottom well plate. In-vitro growth of Kp was estimated by measuring every 15 min for 24 h the optical density at 600 nm on a TECAN Infinite 200 PRO (TECAN) in aerobic conditions.

### Metabolomics analysis

#### Metabolite extraction

Fecal material were pre-extracted and homogenized by the addition of 400 µL of MeOH:H2O (4:1), in the Cryolys Precellys 24 sample Homogenizer (2 × 20 seconds at 10,000 rpm, Bertin Technologies, Rockville, MD, US) with ceramic beads. The bead beater was air-cooled down at a flow rate of 110 L/min at 6 bar. Homogenized extracts were centrifuged for 15 min at 4000 x g at 4°C (Hermle, Gosheim, Germany). The resulting supernatant was collected and evaporated to dryness in a vacuum concentrator (LabConco, Missouri, US). Dried sample extracts were resuspended in MeOH:H2O (4:1, v/v) according to the sample weight.

#### Protein quantification

The protein pellets were evaporated and lysed in 20 mM Tris-HCl (pH 7.5), 4 M guanidine hydrochloride, 150 mM NaCl, 1 mM Na2EDTA, 1 mM EGTA, 1% Triton, 2.5 mM sodium pyrophosphate, 1 mM beta-glycerophosphate, 1 mM Na3VO4, 1 µg/ml leupeptin using the Cryolys Precellys 24 sample Homogenizer (2 × 20 secat 10,000 rpm, Bertin Technologies, Rockville, MD, US) with ceramic beads. BCA Protein Assay Kit (Thermo Scientific, Masschusetts, US) was used to measure (A562nm) total protein concentration (Hidex, Turku, Finland).

#### Data acquisition

Extracted samples were analyzed by Hydrophilic Interaction Liquid Chromatography coupled to tandem mass spectrometry (HILIC – MS/MS)^1,2^ in both positive and negative ionization modes using a 6495 triple quadrupole system (QqQ) interfaced with 1290 UHPLC system (Agilent Technologies). In positive mode, the chromatographic separation was carried out in an Acquity BEH Amide, 1.7 μm, 100 mm × 2.1 mm I.D. column (Waters, Massachusetts, US). Mobile phase was composed of A = 20 mM ammonium formate and 0.1% FA in water and B = 0.1% FA in ACN. The linear gradient elution from 95% B (0–1.5 min) down to 45% B was applied (1.5 min −17 min) and this conditions were held for 2 min. Then initial chromatographic condition were maintained as a post-run during 5 min for column re-equilibration. The flow rate was 400 μL/min, column temperature 25°C and sample injection volume 2 µl. ESI source conditions were set as follows: dry gas temperature 290°C, nebulizer 35 psi and flow 14 L/min, sheath gas temperature 350°C and flow 12 L/min, nozzle voltage 0 V, and capillary voltage 2000 V. Dynamic Multiple Reaction Monitoring (DMRM) was used as acquisition mode with a total cycle time of 600 ms. Optimized collision energies for each metabolite were applied. In negative mode, a SeQuant ZIC-pHILIC (100 mm, 2.1 mm I.D. and 5 μm particle size, Merck, Damstadt, Germany) column was used. The mobile phase was composed of A = 20 mM ammonium Acetate and 20 mM NH4OH in water at pH 9.7 and B = 100% ACN. The linear gradient elution from 90% (0–1.5 min) to 50% B (8–11 min) down to 45% B (12–15 min). Finally, the initial chromatographic conditions were established as a post-run during 9 min for column re-equilibration. The flow rate was 300 μL/min, column temperature 30°C and sample injection volume 2 µl. ESI source conditions were set as follows: dry gas temperature 290°C and flow 14 L/min, sheath gas temperature 350°C, nebulizer 45 psi, and flow 12 L/min, nozzle voltage 0 V, and capillary voltage −2000 V. Dynamic Multiple Reaction Monitoring (dMRM) was used as acquisition mode with a total cycle time of 600 ms. Optimized collision energies for each metabolite were applied.

### Quantification and statistical analysis

Cumulative data are shown as mean±SEM or mean± SD. Statistical tests used include Student’s t-test and Wilcoxon test, Pearson’s correlation. Unless otherwise stated, *p* values < .05 were considered significant. Detailed information on the n of biological replicates and statistical tests used can be found in figure legends. Statistical analyzes were performed using R (v.4.0.3) or GraphPad Prism (v.9.2.0).

### Protein sequence annotation

Protein sequences associated to the coding genes identified by RAST were further annotated with Uniprot database. More specifically, we collected 274,630 unirpot entries associated to the taxonomy of *Klebsiella pneumoniae* (Taxon ID 573) and align our protein sequences to them using NCBI blastp (v2.13.0). Alignments were processed with an R script to retain at most one uniprot entry per input sequence, with the following criteria: 1) only retain alignments with at least 70% identity; 2) only retains alignment where at least 70% of the query and subject sequences are covered; 3) exclude alignments to unreviewed entries if there exists another alignment to a reviewed SwissProt entry; 4) exclude alignment without a primary gene name if there exist another alignment with one; 5) keep the best alignment for each query sequence prioritizing reviewed alignments and highest bit_scores.

## Results

### Rapid and robust transcriptional adaptation of intestinal CR-Kp upon immune response

We previously showed that, in a mouse model of i.p. injection with anti-CD3 antibody, immune activation promotes rapid and robust functional reprogramming of commensal microbes via transcriptional modulation.^34^ Intestinal symbionts adapted to host immune responses within hours, by regulating expression of hundreds of genes, a sizable fraction of which were involved in stress response or utilization of alternative carbon sources. Importantly, we found that transcriptional rewiring of bacteria was associated with dramatic changes in the concentration of luminal metabolites, including decrease in short chain fatty acids (SCFAs) and increase in amino acids and aromatic compounds, all known modulators of host pathophysiology.^34^

**Figure 1.**
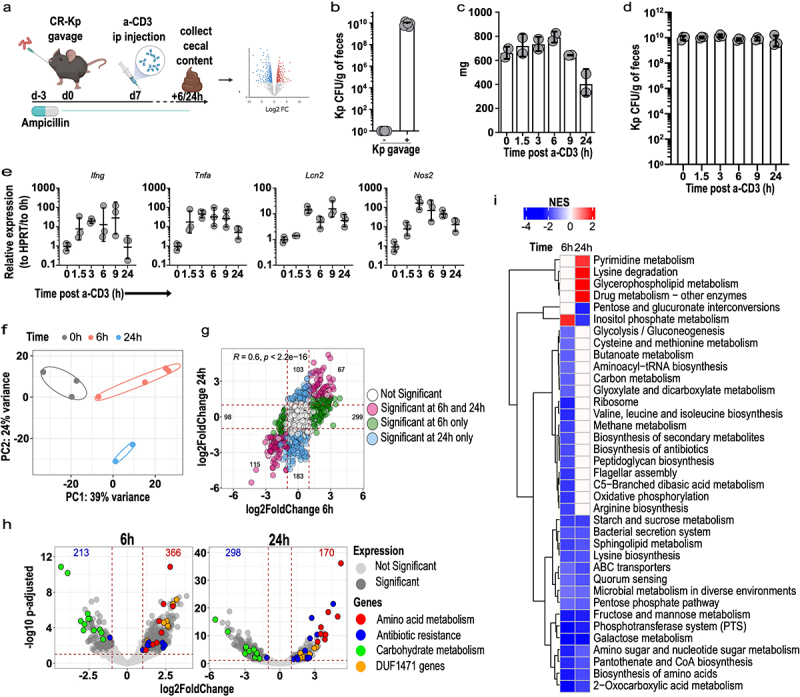
Anti-CD3 treatment modulates transcriptional activity of Carbapenem-resistant *Klebsiella pneumoniae.*

To investigate whether and how immune activation promotes reprogramming of intestinal MDR *Klebsiella pneumoniae*, we reconstituted antibiotic-treated mice with a previously characterized clinical isolate of Sequence Type-258 carbapenem-resistant (KPC) Kp (MH-258, for Memorial Hospital ST-258, hereby CR-Kp)^8,13,35^ (Figure 1a). As reported previously,^12^ CR-Kp reached high densities in the intestine (Figure 1b), without inducing signs of overt inflammation, consistent with the notion that most Kp isolates are avirulent in the gut lumen (data not shown).^21^ One week following reconstitution, we injected animals i.p. with anti-CD3 antibody, which activates T cells producing a systemic cytokine storm and local activation in the intestinal tissue.^36,37^ This model has been widely employed to recapitulate GVHD-induced diarrhea and promotes short-lasting responses and self-limited sickness in the animals^36–39^. We found that the intestinal content was relatively preserved in the cecum over the first 24 h (Figure 1c), while it was ablated in small intestine and colon (data not shown). In the cecum, CR-Kp loads remained constant over time, suggesting that the anti-CD3 treatment does not promote significant bactericidal activity (Figure 1d). As expected, anti-CD3 injection induced expression in the intestinal tissue of genes encoding for inflammatory mediators including IFN-γ, TNF-α, iNos and lipocalin, which were robustly upregulated already 1.5-3 h post-injection (p.i.), and peaked between 3-9 h p.i., decreasing to various degrees over the following 15 h (Figure 1e).

a) Schematic representation of the experimental layout. Mice were treated with ampicillin in drinking water for 3 days and reconstituted with CR-Kp by oral gavage. Seven days later, mice were injected i.p. with anti-CD3 (a-CD3) antibody (100 μg). 6h or 24 h following injection, mice were sacrificed, and RNA was extracted from cecal contents for sequencing.

b) Fecal burden of CR-Kp 24 h after gavage as measured by selective plating.

c) Weight of cecal content collected at the depicted time point following treatment (*n* = 3).

d) CR-Kp CFUs in cecal contents harvested at the indicated times following treatment and measured by selective plating (*n* = 3, one representative of 2 experiments shown).

e) Quantitative PCR for the depicted genes on cecal tissue harvested at the indicated time points following treatment (*n* = 3, shown are geometric means ± SD, one representative of 2 experiments shown).

f) PCA of CR-Kp transcriptome at the indicated time points following treatment (rlog-transformed counts (*n* = 2-4 per group).

g) Correlation plot depicting expression levels for CR-Kp expressed as log2 fold change between expression at 6 h or 24 h post anti-CD3 antibody administration as compared to baseline. Each dot corresponds to a gene, color code indicates: blue = significant DEG only at 24 h; green = significant DEG only at 6 h; pink = DEG significant in both; white = no significant differential expression. Significance was defined as adjusted *p*value < .05 and absolute log2 fold change > 1. Pearson’s correlation coefficient and *p* value are shown.

h) Volcano plot depicting changes in gene expression for CR-Kp at 6 h and 24 h following anti-CD3 antibody treatment, relative to baseline. DEG are shown in dark gray. Selected categories of significant DEG are highlighted as follow: amino acid metabolism = red, antibiotic resistance = blue, carbohydrate metabolism = green, DUF1471-domain protein-encoding genes = orange.

g) Gene Set Enrichment Analysis (GSEA) of the dataset presented in Figure 1f–h. Shown are KEGG categories that were significantly enriched (adjusted *p*value < .05), color represents net enrichment score (NES).

Having confirmed local immune activation in CR-Kp-reconstituted, anti-CD3 treated mice, we conducted RNA-sequencing (RNA-seq) on RNA extracted from their cecal content. Principal component analysis (PCA) revealed a robust shift in CR-Kp transcriptome already 6 h after anti-CD3 treatment, which further evolved at 24 h (Figure 1f). Gene expression profiles were highly concordant at these two time points, possibly indicating persistence of some of the perturbing mediators produced by the treatment (Figure 1g). Gene set enrichment analysis revealed that carbohydrate metabolism (glycolysis/gluconeogenesis, glucose, mannose, starch, sucrose metabolism, as well as phosphotransferase systems, pentose phosphate pathway, amino sugar, and nucleotide sugar metabolism) was strongly and rapidly down-regulated, together with amino acid biosynthesis (Figure 1h).

Analysis at the gene level confirmed that immune activation had produced a very broad and complex rearrangement of CR-Kp transcriptional profile. Hundreds of differentially expressed genes (DEGs) could be identified 6 h (366 up- and 213 down-regulated, respectively) and 24 h post-treatment (170 up- and 298 down-regulated, respectively) (Figure 1i). An in-depth analysis of the top 40 DEGs identified at least four categories of genes, based on function and expression trends, that were over-represented (Figure 1i; for corresponding UniProt annotation of the entries, see Supplementary Table S1). Of note, similar trends of differential expression for analogous categories of genes were previously detected in experiments utilizing a consortium of bona fide commensals, indicating the existence of common strategies across bacterial phyla to cope with the effects of immune activation in the intestinal lumen.^34^ Down-regulated genes, as highlighted by GSEA, were mostly involved in sugar metabolism or amino acid biosynthesis. The phosphotransferase (PTS) system for mannose (peg.4426–4429), glucitol/sorbitol (peg.1533–5), and glucose (peg.4639) were among the top down-regulated genes. Similarly, expression of the genes encoding the maltoporin A/phage lambda receptor protein and the ABC transporter for maltose/maltodextrins (peg.5545–51) was decreased (Figure 1h).

Three groups of genes were on the contrary robustly up-regulated. The first concerned amino acid metabolism and included: a di-tripeptide/H+ symporter *dtpB* (peg.630); serine/threonine degradation operons (peg.2562/2566–1427/1428), critical to promote fitness of *E. coli* in a model of DSS-induced colitis;^20,26^ a lysine/ornithine antiporter and an inducible lysine decarboxylase (peg.4501–4502), implicated in stress response in *Candida albicans;*^40^ an alanine-alanine dipeptide exporter (peg.1568) activated in *E. coli* as a safety valve in conditions of amino acid excess.^41^ Second, we detected genes involved in antibiotic-resistance, including: multidrug resistance protein D (*emrD*) (peg.401), drug resistance transporter *emrB/qacA* subfamily (peg.450), multiple antibiotic resistance protein *marR* (peg.3288), *marA* (peg.3289) and *marB* (peg.3290), multidrug resistance RND efflux system membrane fusion protein *mexE* (peg.1603), multidrug resistance protein *ermA* (peg.1553) and *ermB* (peg.1552). Of note, such up-regulation occurred either preferentially or exclusively 24 h post injection, perhaps indicating over time accumulation of toxic metabolites in the bacterial cells because of immune-mediated perturbations. The third group included genes involved in stress response or host-microbe interaction, most notably genes encoding for proteins containing the domain of unknown function (DUF) 1471. The DUF1471 is a small domain (~70 AA) found across multiple paralogue proteins encoded by *Enterobacteriaceae*, where it is present in 1–3 copies. Some of these proteins have been described to enhance resistance to stressors such as CdCl_2_ or H_2_O_2_, or enable interactions between the bacterium and the host.^42^ The above findings were confirmed in an independent RNAseq experiment and further validated by qPCR (Supplementary Figure S1a-c).

Taken together, our data indicate that immune activation or its consequences on luminal metabolites promotes rapid, complex, and well-defined transcriptional rewiring in intestinal CR-Kp, possibly affecting its metabolism (decrease sugar utilization, increased amino acid catabolism), resistance to stressors and antibiotics, or interaction with host cells.

### Identification of an immune-dependent CR-Kp transcriptional signature conserved across vivaria and microbiota configuration

**Figure 2.**
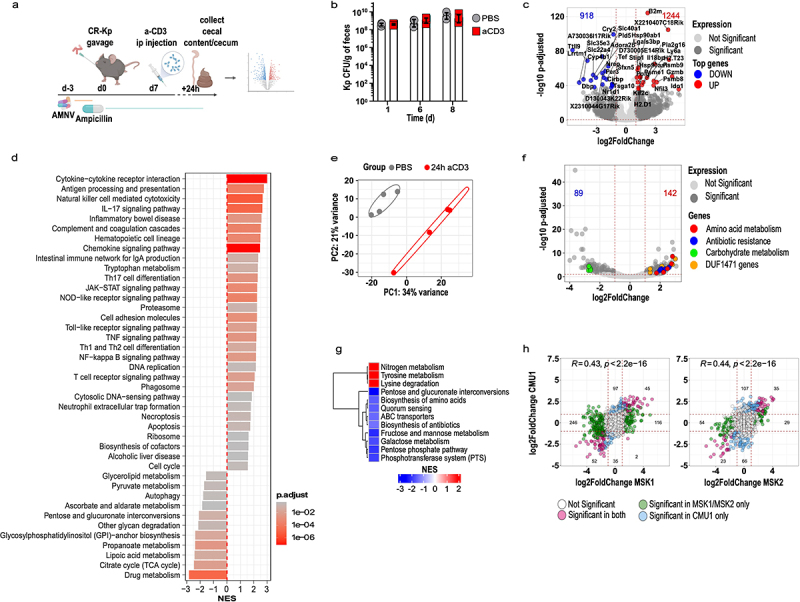
A dual-seq experiment validates mouse and CR-Kp transcriptional shifts following immune activation.

Differences across animal facilities are known to reduce reproducibility of findings in studies involving gut microbes, primarily because of the distinct microbial configurations hosted by the respective animals. We took advantage of the relocation of our laboratory to a different institution to determine a reliable signature of immune-dependent CR-Kp transcriptional rewiring. In the new vivarium, mice harbored a microbiota that resulted largely resistant to ampicillin (data not shown). To deplete the endogenous flora, we therefore employed a broader-spectrum antibiotic regimen, namely a combination of ampicillin, metronidazole, neomycin, and vancomycin (AMNV), followed by gavage with CR-Kp and ad libitum treatment with ampicillin (Figure 2a). In these settings, CR-Kp could colonize the intestine, albeit to lower densities than those reached previously (~10^8-^10^9^ vs ~ 10^10^ CFUs/g of feces) (Figure 2b, compare with Figure 1b).

a) Schematic representation of the experimental layout. Mice were treated with AMNV (A = Ampicillin, *M* = Metronidazole, *N* = Neomycin, V = Vancomycin) and reconstituted with Kp for 7 days before getting injected i.p. with anti-CD3 antibody (100 μg). RNA was extracted from cecal content and tissue and sequenced (*n* = 4 per group).

b) Fecal burden of CR-Kp at the depicted time points following treatment, as measured by selective plating.

c) Representative volcano plot depicting mouse DEG at 24 h post anti-CD3 antibody treatment (as compared to time-matched PBS treatment). Genes DE are shown in dark gray. Non-DE are shown in light gray. Top 40 DE genes are highlighted as follow: red = 20 most up-regulated genes, blue = 20 most down-regulated genes.

d) GSEA of murine RNAseq data set. Shown are KEGG categories that were significantly enriched (adjusted *p* value < .05, color-coded), bars represents net enrichment score (NES).

e) PCA of CR-Kp transcriptome 24 h following treatment (rlog-transformed counts (*n* = 4 per group).

f) Volcano plot depicting changes in gene expression for CR-Kp 24 h following anti-CD3 antibody treatment, relative to time-matched PBS-treated controls. DEG are shown in dark gray. Selected categories of significant DEG are highlighted as follow: amino acid metabolism = red, antibiotic resistance = blue, carbohydrate metabolism = green, DUF1471-domain protein-encoding genes = orange.

g) (GSEA) of the dataset presented in Figure 1f–h. Shown are KEGG categories that were significantly enriched (adjusted *p* value < .05), color represents net enrichment score (NES).

h) Correlation plot depicting expression levels for CR-Kp expressed as log2 fold change between expression 24 h post anti-CD3 antibody administration as compared to baseline, in experiments MSK1 vs CMU1 and MSK2 vs CMU1. Each dot corresponds to a gene, color code indicates: blue = significant DEG only in CMU1; green = significant DEG only in MSK1/MSK2; pink = DEG significant in both; white = no significant differential expression. Significance was defined as adjusted *p* value < .05 and absolute log2 fold change > 1. Pearson’s correlation coefficient and *p* value are shown.

To obtain information about bacterial and host transcriptional rearrangements, we performed sumultanous RNA sequencing (Dual-Seq)^43^ on RNA extracted from cecal content and tissue of mice reconstituted with CR-Kp and treated with PBS or anti-CD3 24 h prior. Analysis of the mouse transcriptome revealed substantial up-regulation of immune-related genes, including many interferon-regulated genes (*Gbp2-6-10, CXCL9, Ly6A, Igtp, MHC*), T cell effector molecules (*Gzma, Gzmb*), cytokines and enzymes involved in inflammatory responses (*Tnf, Ido1, Nos2, Lcn2*) to a comparable extent to what observed in our initial experiments (Figure 2c). GSEA confirmed up-regulation of multiple immune-related pathways and identified down-regulation of several carbon metabolism pathways, including pyruvate metabolism, pentose and glucuronate interconversion, TCA cycle, and glycan degradation (Figure 2d).

On the bacterial side, we detected hundreds of up- and down-regulated genes in CR-Kp from anti-CD3 treated animals, which belonged to the functional categories identified in our previous experiments (Figure 2e, f). In agreement with our initial findings, GSEA highlighted down-regulation of PTS, pentose phosphate, fructose and mannose metabolism, and biosynthesis of amino acids, while degradation of amino acids was enhanced (Figure 2g). Furthermore, individual gene expression levels robustly and significantly correlated across different experiments (Figure 2h).

**Figure 3.**
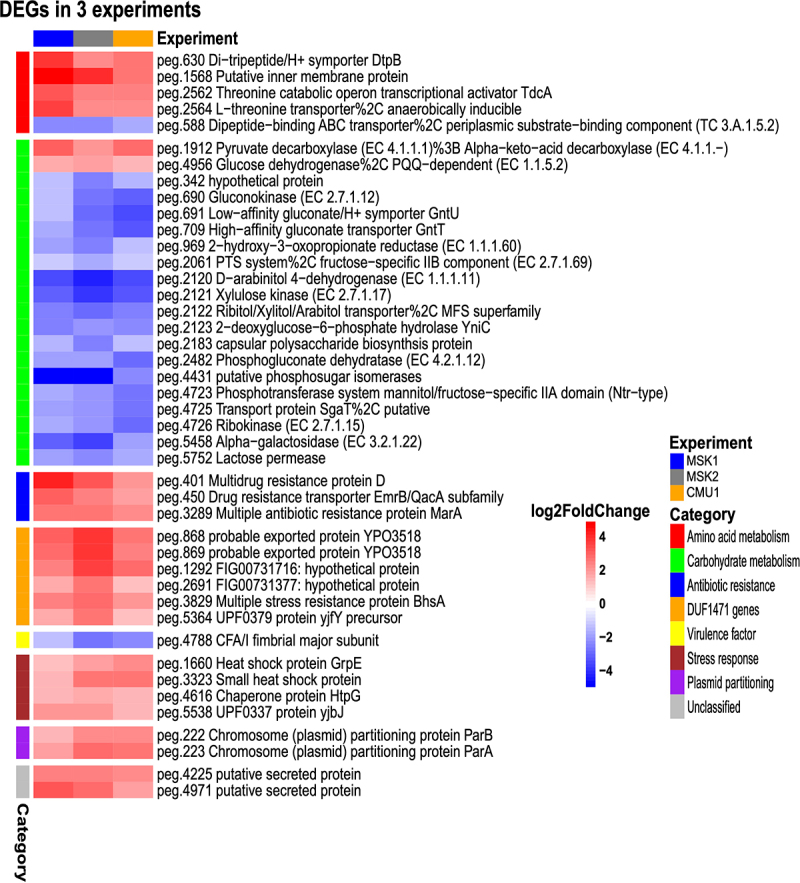
Identification of a robust transcriptional signature of CR-Kp adaptation to intestinal immunity.

Notably, aggregate PCA revealed that samples clustered primarily on the basis of the experiment, indicating that experiment-dependent variables (likely the nature of the residual microbiota) may produce substantial variability in the baseline transcriptome of CR-Kp (Supplementary Figure S2). However, upon anti-CD3 treatment, a concordant shift along the second component axis occurred across experiments (Supplementary Figure S2). Thus, we integrated the three datasets to identify a common transcriptional signature that may be induced regardless of environmental variables. We found that 40 genes were consistently differentially regulated across experiments (padj <0.05, absolute (log2FC) >1), most of which ranked amongst the top DEG and belonged to the functional categories discussed above (Figure 3). Furthermore, 127 additional genes were significantly differentially expressed in two out of three experiments, and showed mostly comparable modulation patterns across the three experiments, suggesting that the conserved signature may actually be broader (Supplementary Figure S3).

(a) Heatmap depicting log 2-fold change expression levels for genes concordantly up- or down-regulated by CR-Kp in response to immune activation in three different experiments (MSK1, MSK2, CMU1) carried out in 2 different *vivaria*. Functional annotation is shown on the left and it is based on PATRIC subsystem, gene level. Category annotation was curated manually.

Overall, we identified multiple genes that are rapidly and consistently regulated in intestinal CR-Kp following immune activation and may modulate bacterial fitness or interaction with the host under inflammatory conditions.

### Oxidative species drive expression of DUF1471-encoding genes

To begin investigating the functional relevance of the above signature, we decided to focus DUF1471-encoding genes, which were rapidly and strongly induced by immune activation (Figures 1h, 2f, 3).

A conserved domain search (https://www.ncbi.nlm.nih.gov/Structure/cdd/wrpsb.cgi) identified 13 proteins encompassing a DUF1471 domain in the employed CR-Kp strain (peg.713, peg.868, peg.869, peg.1292, peg.2691, peg.3406, peg.3504, peg.3829, peg.4153, peg.4155, peg.4504, peg.5364, peg.5376). Six hour post injection, 6–7 of these were consistently found within the top 50 up-regulated genes (significant enrichment as per hypergeometric test, *p* value~ 9 × 10^−11^). This observation suggested that expression of these genes may be coordinated and potentially relevant for bacterial fitness in the inflammatory milieu.

These genes were predicted to encode proteins of reduced length (61–91 amino acids) which displayed very little sequence similarity, despite the common presence of a DUF1471 domain (Supplementary Figure S4a). All the predicted proteins contained a signal peptide, suggesting localization either in the periplasm or in the extracellular space (ProtCompB, Softberry, Inc) (Supplementary Table S2). Based on homology (Supplementary Table S1), peg.868 and peg.869 were annotated as YhcN, a stress-induced protein that promotes resistance to CdCl_2_ or H_2_O_2_.^44^ Peg.3829 corresponds to multiple stress resistance protein BhsA, which promotes survival to acid, heat treatment, hydrogen peroxide, and cadmium and negatively regulate inter-bacterial adhesion and biofilm.^45^ Peg.1292 was reported to decrease Kp adhesive capacity by negatively regulating type III fimbriae^46^ but also to reduce capsule and biofilm formation.^47^ Peg.4155 encodes Yibj, which can promote biofilm formation and motility.^48^ Finally, peg.5364 encodes the YjfY protein, which has not been characterized. However, a STRING analysis revealed that Yibj is connected to BhsA (peg.3829), YhcN (peg.868/9), and YbiJ (peg.4155) in *E. coli* based on co-occurrence and text mining (Supplementary Figure S4b).

**Figure 4.**
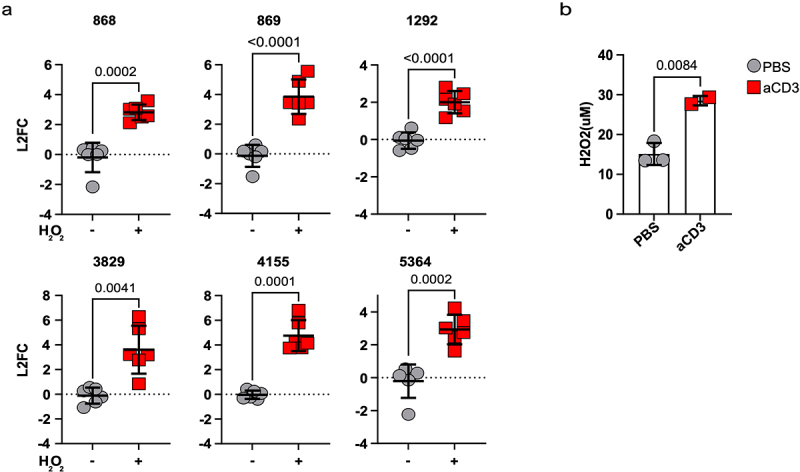
DUF1471 protein-encoding genes can be up-regulated by reactive oxygen species.

As several of these genes had been previously shown to promote resistance to H_2_O_2_, we asked if this molecule could promote their expression. Indeed, exposing CR-Kpn to H_2_O_2_ in culture, resulted in rapid up-regulation of these genes (Figure 4a). Notably, i.p. injection of mice with anti-CD3 antibody also resulted in rapid accumulation of H_2_O_2_, suggesting that release of oxidizing agents could promote or contribute to up-regulation of DUF1471-encoding genes *in vivo* (Figure 4b).

(a) Quantitative PCR for the depicted genes performed on WT Kp harvested at 30 min following exposure to sub-lethal concentrations of H_2_O_2_ (i.e. 1.25 mM) (*n* = 6 from 3 independent experiments, shown are means ± SD, Multiple unpaired t-test with Welch’s correction, *p* < .05).

(b) Quantification of H_2_O_2_ in the cecum of WT mice measured with a colorimetric assay at the depicted time point post-injection of anti-CD3 (*n* = 2-3 per group; shown 1 representative of 2 experiments; unpaired t-test).

### Cryptic function of DUF1471-engoding genes

To address a potential role for the above genes in regulating bacterial fitness in response to H_2_O_2_ or other stressors, we generated individual isogenic deletion mutants and tested their survival through *in vitro* oxidative stress assays but observed no defect as compared to WT Kp (data not shown). Given the presence of a common domain in the proteins encoded by the above genes, we reasoned that despite the little homology displayed they may possess redundant functions, so that the absence of one member could be functionally compensated by the presence of others.

**Figure 5.**
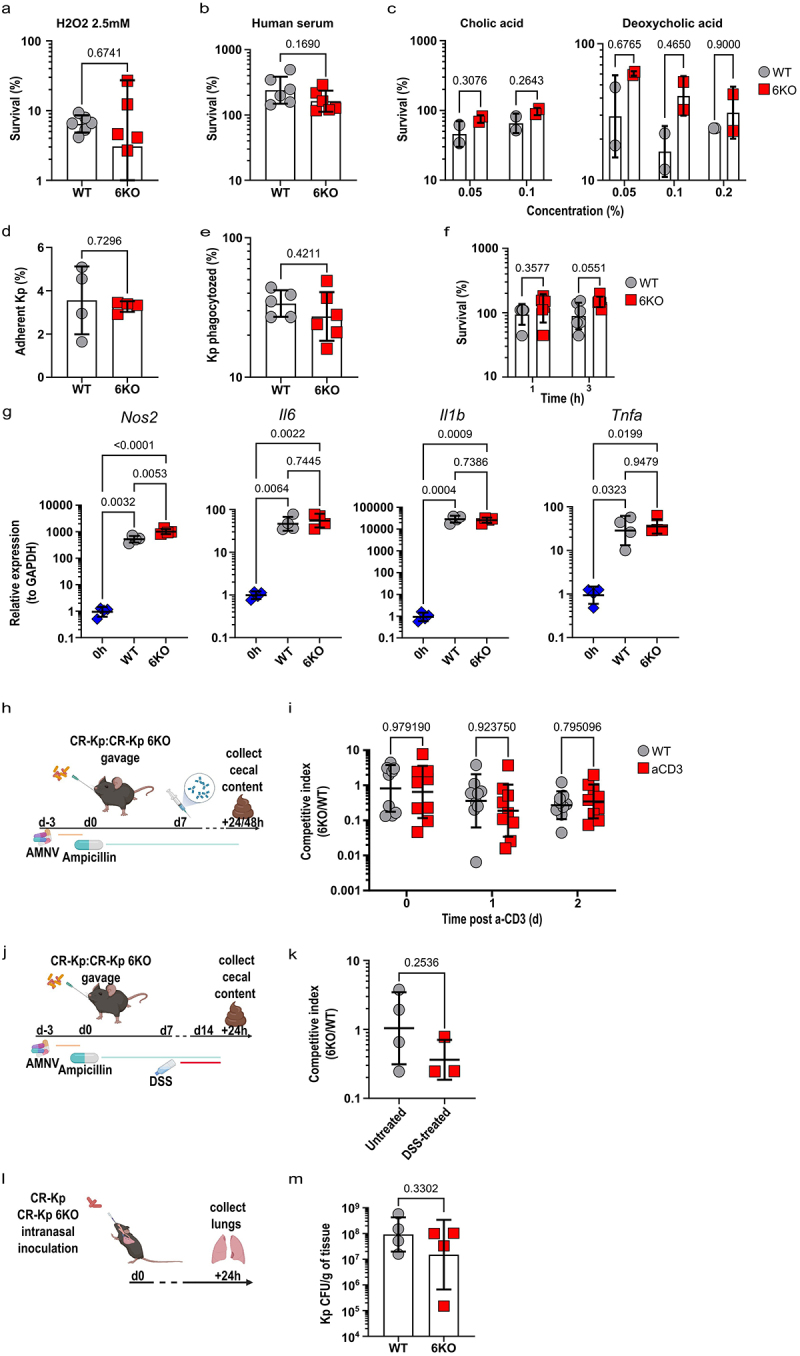
Absence of 6 DUF1471 protein-encoding genes does not affect the fitness and the virulence of *Klebsiella pneumoniae* in-vitro and in-vivo.

To account for this possibility, we generated a scarless mutant strain lacking 6 DUF1471-encoding genes of interest (hereby referred to as ‘6KO’) using a recently published CRISPR-based approach^49,50^ (Supplementary Figure S5). To our surprise, *in vitro* survival of the 6KO CR-Kp was comparable to that of WT CR-Kp in response to several broadly utilized bacterial stressors, including H_2_O_2_, human serum, and bile acids (Figure 5a–c). Similarly, when testing acidic stress resistance, biofilm formation or surface hydrophobicity, which were other reported roles for individual DUF1471-encoding genes,^44–48,51^ we detected no difference between 6KO and WT CR-Kp (data not shown).

(a) Survival of the indicated CR-Kp strains exposed to 2.5 mM H_2_O_2_ for 1 h. Survival is expressed as percentage of recovered CFUs relative to initial inoculum (*n* = 6 from 2 independent experiments, shown are geometric means ± SD; unpaired t-test, *p* < .05).

(b) Survival of the indicated CR-Kp strains exposed to human serum for 1 h. Survival is expressed as percentage of recovered CFUs relative to initial inoculum (*n* = 6 from 2 independent experiments, shown are geometric means ± SD; unpaired t-test, *p* < .05).

(c) Survival of the indicated CR-Kp strains grown overnight at 37°C on LB-agar plates supplemented with cholic acid (0.05%, 0.1%) or deoxycholic acid (0.05%, 0.1%, 0.2%). Survival is expressed as percentage of recovered CFUs relative to initial inoculum (*n* = 2, shown are geometric means ± SD; 2-way ANOVA, *p* < .05).

(d) Proportion of Kp adherent to HEK293T cells after 1 h incubation at 37°C. Proportion is expressed as percentage of CFUs recovered after extensive cell wash, relative to initial inoculum (*n* = 4, shown are means ± SD; unpaired t-test, *p* < .05).

(e) Proportion of Kp phagocytosed by RAW264.7 macrophages after 1 h incubation. Proportion is expressed as percentage of CFUs recovered after extensive cell wash and gentamycin treatment, relative to initial inoculum (WT, *n* = 5, 6KO *n* = 6, shown are geometric means ± SD; unpaired t-test, *p* < 0.05).

(f) Survival of the indicated Kp strains phagocytosed RAW264.7 macrophages for 1 h or 3 h post gentamicin wash at 37°C. Survival is expressed as percentage of the CFUs recovered after incubation, as compared to cells phagocytosed at *t* = 0 (*n* = 6, shown are geometric means ± SD; 2-way ANOVA, *p* < .05).

(g) Quantitative PCR for the depicted genes on RAW264.7 macrophages harvested at the indicated times following exposure to CR-Kp (*n* = 4, shown are geometric means ± SD, 1-way ANOVA, *p* < .05).

(h) Experimental layout. Mice were treated with AMNV (A = Ampicillin, *M* = Metronidazole, *N* = Neomycin, V = Vancomycin) and reconstituted with a consortium consisting of a 1:1 ratio of WT:6KO CR-Kp for 7 days before getting injected i.p. with anti-CD3 (a-CD3) antibody (100 μg). Bacterial genomic DNA was extracted from cecal contents of animals reconstituted.

(i) Quantitative PCR with strain-specific primers was performed on the DNA obtained in (h) to calculate the competitive index between the WT Kp and the 6KO Kp at the indicated time points (*n* = 9 from 2 independent experiments, shown are geometric means ± SD, 2-way ANOVA, *p* < .05).

(j) Experimental layout. Mice were treated with AMNV (A = Ampicillin, *M* = Metronidazole, *N* = Neomycin, V = Vancomycin) and reconstituted with a consortium consisting of a 1:1 ratio of WT:6KO CR-Kp for 7 days before getting treated with Dextran Sodium Sulfate (DSS) (3%) for 7 days. Bacterial genomic DNA was extracted from cecal contents of animals reconstituted.

(k) Quantitative PCR with strain-specific primers was performed on the DNA obtained in (J) to calculate the competitive index between the WT Kp and the 6KO Kp at the indicated time points (*n* = 4, shown are geometric means ± SD, unpaired t-test, *p* < .05).

(l) Experimental layout. Mice were intranasally inoculated with either WT or 6KO CR-Kp. 24 h post inoculation, CR-Kp burden in the lung tissue was determined by selective plating and CFU enumeration.

(m) Pulmonary levels of WT and 6KO CR-Kp obtained from (l) (*n* = 4, shown are geometric means ± SD, unpaired t-test, *p* < .05).

DUF1471-genes can modulate interaction with host cells.^42,45,52^ However, 6KO CR-Kp mutants showed no defect in cell adhesion and were as resistant as the parental strain to macrophage killing (Figure 5d–f). Consistent with this observation, we found that the activation profile promoted by WT and 6KO CR-Kp in macrophages was comparable and encompassed strong up-regulation of inflammatory genes such as *Il1b, Il6*, and *Tnfa* (Figure 5g). In two independent experiments, we detected a minimal, albeit significant, increase in the induction of *Nos2* by 6KO CR-Kp, which we deemed irrelevant (Figure 5g and data not shown).

Competition assays carried out *in vivo*, in mice reconstituted with 1:1 ratios of WT and 6KO CR-Kp and treated with anti-CD3 or DSS to promote acute or sustained intestinal inflammation, revealed only non-significant trends toward a loss of fitness in the mutant strain (Figure 5h–k). Similarly, DUF1471 gene products where not required for CR-Kp to withstand immune insults in a model of lung infection (Figure 5l, m). The function of the DUF1471-bearing proteins was not masked by the overarching protection provided by the capsule, as capsule deletion^13^ in WT and 6KO CR-Kp did not uncover any difference between the two strains in the above *in vitro* and *in vivo* assays (Supplementary Figure S6a-l).

Thus, while robust up-regulation of DUF1471-encoding genes occurs in the gut lumen of immune-activated mice, as a result of oxidative stress or other inflammation-derived signals, the function of the encoded proteins in the studied CR-Kp strain remains cryptic.

### Broad shifts in luminal metabolome following anti-CD3 treatment

The above results suggested that stress-response factors may be redundant in function, making it difficult to address their individual role in the utilized model. Following immune activation, genes involved in carbon metabolism were also strongly modulated. We reasoned that investigating the metabolic rewiring occurring in CR-Kp, which potentially reflects shifts in carbon source availability, may provide alternative strategies to interfere with adaptation of the bacterium to the intestinal milieu.

**Figure 6.**
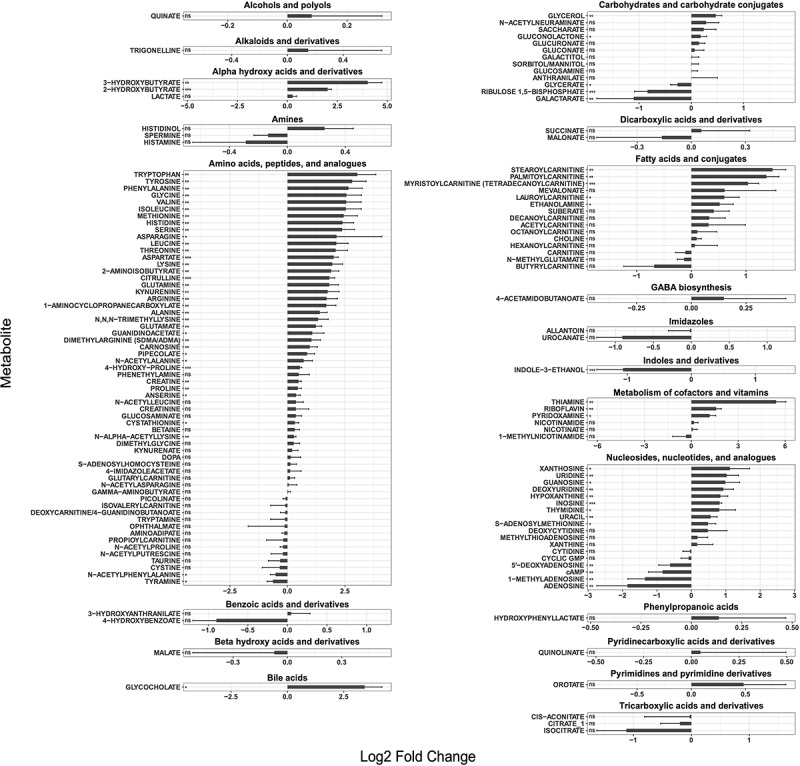
Major metabolic shifts are observed in the gut lumen during inflammation.

To this aim, we performed a metabolomics analysis of the cecal content from mice 5 hours following PBS or anti-CD3 treated treatment. For this analysis, we utilized mice treated with broad-spectrum antibiotics and confirmed to be microbiota-depleted by plating (Supplementary Figure S7), so to minimize the impact of bacteria-derived metabolites. We detected very significant changes in the metabolic landscape of the cecum, including a strong increase in the concentration of amino acid, which is consistent with our previously published data obtained in SPF mice,^34^ as well as with the transcriptional signature detected in CR-Kp (up-regulation of amino acid import and catabolism, and down-regulation of amino acid biosynthesis) (Figure 6). Notable shifts were observed for many different classes of molecules, and included a strong increase in bile acids, nucleotides and nucleosides, vitamins, and cofactor (particularly thiamine), and also fatty acids (Figure 6 and data not shown). Of note, we were unable to detect monosaccharides such as glucose, fructose and mannose, in the cecum of both PBS and anti-CD3 treated mice. Similar results were obtained via stimulation of animals with distinct immune agonists, indicating that the described metabolic switches likely represent a common outcome of different immune responses (data not shown). Thus, immune activation profoundly remodels the metabolic landscape in the intestinal lumen, possibly changing availability of carbon sources, and triggering rapid adaptation of CR-Kp.

Barplot depicting the levels of polar metabolites detected in the intestinal lumen expressed as concentration fold change in the cecal content of anti-CD3 vs PBS-injected animals (at 5 h). Each bar corresponds to one metabolite(shown are means ± SD; 1-way ANOVA, ns *p* > .05, *p ≤ .05, **p ≤ .01, ***p ≤ .001).

### CRP may be an important modulator of CR-Kp transcriptional adaptation, and is required for intestinal colonization

**Figure 7.**
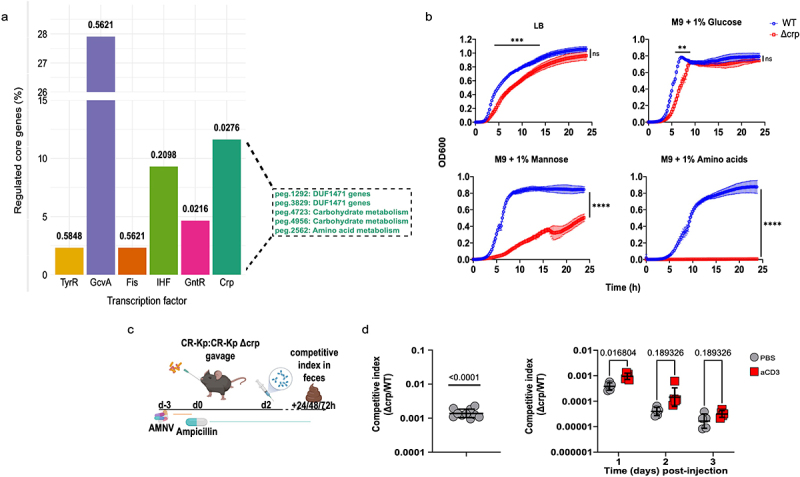
CRP is an important regulator of CR-Kp intestinal colonization but is dispensable for adaptation to inflammation.

The broad transcriptional shifts occurring in CR-Kp following immune activation, and the striking differences detected in concentration of metabolites, including potential carbon sources, prompted us to investigate whether a global regulator may direct the pathobiont’s multifaceted rewiring. We performed a bioinformatic analysis utilizing MEME suite (version 5.5.4) to identify transcription factors potentially responsible for the transcriptional signature depicted in Figure 3. This analysis predicted several transcription factors that could theoretically regulate a sizable fraction of such core signature (Figure 7a). Of these, only two resulted statistically significant, and the cAMP Receptor Protein (CRP) was the one regulating the highest number of genes. CRP is known to modulate both carbon response (i.e. switch to the utilization of distinct carbon sources, including amino acids, in the absence of glucose) as well as stress response and virulence.^53–55^ Accordingly, some DUF1471 genes as well as Serine/Threonine hydratase were predicted to be regulated by this transcription factor (Figure 7a). To test the relevance of CRP in the utilized assays, we generated an isogenic mutant lacking this gene. Δ*crp* CR-Kp could grow similarly to WT CR-Kp in LB and minimal medium M9 supplemented with glucose, displaying slightly delayed kinetics (Figure 7b). However, the strain’s growth was severely impaired and completely abolished when mannose or amino acids were used, respectively, as the sole carbon source (Figure 7b), consistent with the role of CRP in directing metabolic shift to alternative carbon sources.

(a) Barplot depicting the percentage of core differentially expressed genes regulated by the top enriched transcription factors, based on adjusted p-value. On the right are showed the core differentially expressed genes regulated by CRP and their affiliated metabolism (hypergeometric test, *p* < .05).

(b) In-vitro growth of Δcrp Kp and WT Kp were measured via optical density reading every 15 min for 24 h in aerobic conditions (shown are means ± SD, Multiple unpaired t-test with Welch’s correction, ns *p* > .05, * *p* ≤ .05, ** *p* ≤ .01, *** *p* ≤ .001, **** *p* ≤ .0001).

(c) Schematic representation of the experimental layout. Mice were treated with AMNV (A = Ampicillin, *M* = Metronidazole, *N* = Neomycin, V = Vancomycin) and reconstituted with Kp for 2 days before getting injected i.p. with anti-CD3 antibody (100 μg). DNA was extracted from fecal pellets just before injection and at 24 h, 48 h and 72 h post-injection (*n* = 5 per group).

(d) Competitive index between Δcrp and WT Kp as measured by qPCR in feces at the indicated time points following co-administration of the 2 strains via oral gavage. Left: competitive index 24 h post-gavage (*n* = 10, shown are geometric means ± SD, one-sample t-test vs theorical value of 1.0). Right: competitive index at the indicated time points post PBS or anti-CD3 injection (*n* = 5 from 1 representative experiment of 2 performed, shown are geometric means ± SD, Multiple unpaired t-test with Welch’s correction, *p* < .05).

Next, we tested whether CRP might be responsible for adaptation to immune activation *in vivo*, using a competitive index assay (Figure 7b). Importantly, we found Δ*crp* CR-Kp to be severely defective in colonization of the gut, both in the context of competitive index, as well as in that of individual reconstitution, suggesting that this transcription factor is crucial for *Klebsiella* to adapt to the carbon sources available in the intestinal lumen (Figure 7c left, and data not shown). Anti-CD3 treatment did not further impair the mutant’s fitness, indicating that CRP may not contribute to adaptation to metabolites released upon immune activation or, alternatively, that the inherent defect caused by CRP deficiency may overshadow deficits in the adaptation to the inflamed milieu (Figure 7c, right).

## Discussion

MDR bacterial infections are a global health threat, and the WHO has included them in the list of top-10 worldwide healthcare priorities. A recent meta-analysis estimated that roughly 600’000 deaths were caused by antibiotic-resistant *Klebsiella pneumoniae* in 2019 and that almost half of these were directly attributable to the resistance mechanism, i.e. they would not have occurred had the pathogen responded to treatment.^56^

The major reservoir of MDR Kp is the intestinal tract. A sizable fraction of individuals carries low amounts of intestinal MDR-Kp, which blooms following antibiotic treatment mostly in healthcare settings, facilitating infection and transmission to other patients.^8–10,57^ Several mouse models have been established to recapitulate intestinal MDR-Kp colonization and identify the underlying mechanisms of adaptation and transmission;^6,8,12,13^ however, the interplay between the bacterium and host immunity in this context has not been studied. Dissecting the adaptation of Kp to immune cues in the intestine is particularly relevant, as this bacterium is enriched in the stools of IBD patients, and experiments carried out in animal models have confirmed that some Kp strains can promote enteric inflammation.^21–23–27–58^

Here, using an established model of acute immune activation,^34^ we found that carbapenem-resistant (CR)-Kp underwent sustained shifts in gene expression as early as 6 hours and for at least 24 h following the initial immune stimulus. We could confirm modulation of genes and pathways impacted by such rapid adaptation across experiments performed in distinct *vivaria* and following different antibiotic treatments. The detected transcriptional shifts underlain an overall metabolic rewiring, including strong down-regulation of sugar metabolism (PTS system, metabolism of galactose, fructose, mannose, starch and sucrose, xylose) and amino acid synthesis, and promotion of amino acid import and degradation (threonine catabolic operon transcriptional activator TdcA, threonine and serine transporters and dehydratases). In agreement with these transcriptional data, we detected heightened luminal concentrations of amino acid following anti-CD3 treatment via metabolomics analyzes. Such transcriptional and metabolic shifts are in line with what we previously reported for commensal bacteria^34^ and others described for *E. coli* in a model of DSS-induced colitis.^20^

Of note, metabolomics analyses were carried out in microbiota-depleted mice, suggesting that amino acid accumulating in the gut should be of host origin, although a definitive proof will require experiments in germ-free mice. Whether the increase in luminal concentrations of amino acids following immune activation is caused by enhanced protein degradation, active release, or reduced absorption, remains to be determined. However, this phenomenon appears to be a hallmark of intestinal inflammation in animal models^20^ and human patients.^59,60^

*In vitro*, amino acids that are inexpensive to produce such as serine, glycine, threonine, and aspartate, when present in sufficient amounts, can be taken up by *E. coli* and catabolized rather than used as building blocks to ensure faster growth even in the presence of glucose. Accumulation of amino acid degradation metabolites such as pyruvate and oxaloacetate can directly inhibit the phosphotransferase system (PTS).^61^ As we could not detect glucose in the cecal content of antibiotic-treated mice, we speculate that upon inflammation CR-Kp may be taking up amino acids that suddenly become more available, and that the shutdown of PTS/sugar metabolism may be a downstream consequence. Interestingly, PTS gene expression inversely correlates with capsule proficiency in *Klebsiella* strains, and deletion of an glucose PTS system EIIA gene enhanced transcription of capsular enzymes promoting production of a thicker capsule and augmenting resistance to hydrogen peroxide *in vitro*.^62^ Thus, while the above metabolic rewiring may simply reflect changes in carbon sources upon inflammation in the gut, it may also represent an evolutionary strategy to promote fitness in the host in the presence of an immune threat.

We detected consistent and robust up-regulation of several genes encoding the domain of unknown function 1471 (DUF1471) following immune activation. Proteins containing this domain have been described in other *Enterobacteriaceae* (including *E. coli* and *Salmonella* spp.) and can be grouped into several families, whose paralog members have been described to modulate stress responses, adhesion to host or biofilm formation. Expression of some of these genes is regulated by the cyclic AMP receptor protein (CRP).^46^ These genes were previously shown to enhance bacterial fitness and survival in stress conditions such as exposure to H_2_O_2_ across multiple studies, mostly in *E. coli*.^44,45,51^ This potential role seems consistent with our findings that H_2_O_2_ concentrations increase in the intestinal lumen following anti-CD3 treatment and that H_2_O_2_ triggers DUF1471 gene up-regulation *in vitro*. To our surprise, neither single isogenic *Klebsiella* mutants nor a mutant strain lacking 6 relevant DUF1471 genes displayed fitness defects *in vitro*, upon exposure to stressors or cells, or *in vivo* in models of colonization and infection. It is possible that such discrepancy depends on the bacterial species and strains utilized in different studies. Further investigations will be required to verify the role of this shared domain, which may confer proteins some common functional features. Our results also raise the possibility that a network of effector genes may be involved in promoting adaptation of *Klebsiella pneumoniae* to the inflamed intestine, and that deletion of individual factors may not be sufficient to impair bacterial fitness in these settings.

Motif recognition analyses identified the cAMP-Receptor Protein (CRP) as a potential regulator of the described adaptation signature. CRP is a known regulator of the carbon response, i.e. switch to alternative carbon source metabolism in the absence of glucose. Further, CRP has been implicated in the regulation of multiple stress and virulence factors, together with antibiotic resistance.^53–55, 63–65^Using an isogenic Δ*crp* mutant we could not observe, at least in the tested experimental conditions, any role for this gene in modulating fitness in an immune-activated scenario; however, we report that *crp* is *per se* crucial to establish colonization of the gut in the first place. This is in keeping with our failure to detect glucose in the mouse intestine via metabolomics approaches. It is still possible that CRP plays a role in adaptation to inflammatory conditions, but that such effect is overshadowed by the stronger fitness defect on colonization, therefore further experiments will be necessary to obtain a definitive answer to this question.

Our data confirm previous findings in other *Enterobacteriaceae* and greatly expand our understanding of how *Klebsiella* adapts to the intestinal environment and its perturbations. *E. coli*, a widely studied member of *Enterobacteriaceae* which is closely related to Kp, was previously shown to transcriptionally adapt to inflammation in different mouse models of colitis, including DSS treatment^20^ and IL-10^−/−^ mono-association.^66^ In these scenarios, *E. coli* was found to down-regulate genes involved monosaccharide-utilization^20^ and up-regulate genes promoting stress-response, iron-metabolism and amino acid catabolism.^20,66^ *YcfR* (*Bhsa*), corresponding to our peg.3829, was also upregulated in this dataset.

The findings described in our study may have important implications for therapeutic approaches aimed at diminishing the density of *Klebsiella pneumoniae* in the gut of patients. In fact, while the study of these genes was not the object of the current work, we identified multiple genes of potential therapeutic relevance as to be modulated by immune activation. The phage lambda receptor Maltoporin, was significantly and strongly downregulated following anti-CD3 treatment in our experiments, possibly corroborating the notion that perturbations of the intestinal milieu can affect sensitivity to phage infection by regulating gene expression.^67^ Our results suggest therefore that the immune status of a host may impair the efficacy of phage therapy, at least in the intestinal lumen. Genes encoding drug efflux pumps were also modulated, particularly at later time points (24 h), which might provide resistance to toxic metabolites accumulating inside the cell as the immune response progresses. For instance, we observed up-

Genes encoding drug efflux pumps were also modulated, particularly at later time points (24 h), which might provide resistance to toxic metabolites accumulating inside the cell as the immune response progresses. For instance, we observed up-regulation of the *mexE/mexF* efflux system, known to extrude antibiotic molecules of multiple classes in *Pseudomonas aeruginosa* and *E. coli*;^68,69^ multidrug resistance protein D (*emrD*) which belongs to the major facilitator superfamily (MFS) superfamily,^70^ and is supposed to extrude uncouplers (e.g. benzalkonium, SDS), but also arabinose;^71^ multiple antibiotic resistance protein *MarA*, a transcription factor belonging to the AraC/XylS family of regulators which indirectly promotes expression of the *acrAB* genes encoding a multidrug efflux pump. Thus, our findings suggest that sensitivity of luminal Kp ST258 to antibiotics may vary depending on immune status, which could be relevant in clinical scenarios in which *Klebsiella* intestinal carriage represents a risk factor, and occurs either in the presence or absence of intestinal inflammation.

In conclusion, we found that CR-Kp capacity to colonize the intestine requires CRP and that once colonization is established the bacterium can rapidly sense immune activation likely through changes in available carbon sources and accumulation of stressors such as oxidative species and adapt by modulating genes involved in the relative pathways. Activated pathways are likely to be highly redundant, as targeted deletion of key genes involved in the resulting transcriptional signatures did not impair bacterial fitness in inflammatory conditions.

This information may provide guidance to design future strategies to tackle CR-Kp intestinal overgrowth and interaction with immune responses.

## Supplementary Material

Supplemental Material

## Data Availability

The data that support the findings reported in this study are openly available on Yareta [DOI: 10.26037/yareta:qbe6nnbwlbg2ne7ob43cbdkklq].
