# Supplementary figures and images for: Intestinal carbapenem-resistant Klebsiella pneumoniae undergoes complex transcriptional reprogramming following immune activation
Source: Gut Microbes. 2024 Apr 24;16(1):2340486. doi: 10.1080/19490976.2024.2340486 (PMC11057644; doi:10.1080/19490976.2024.2340486)

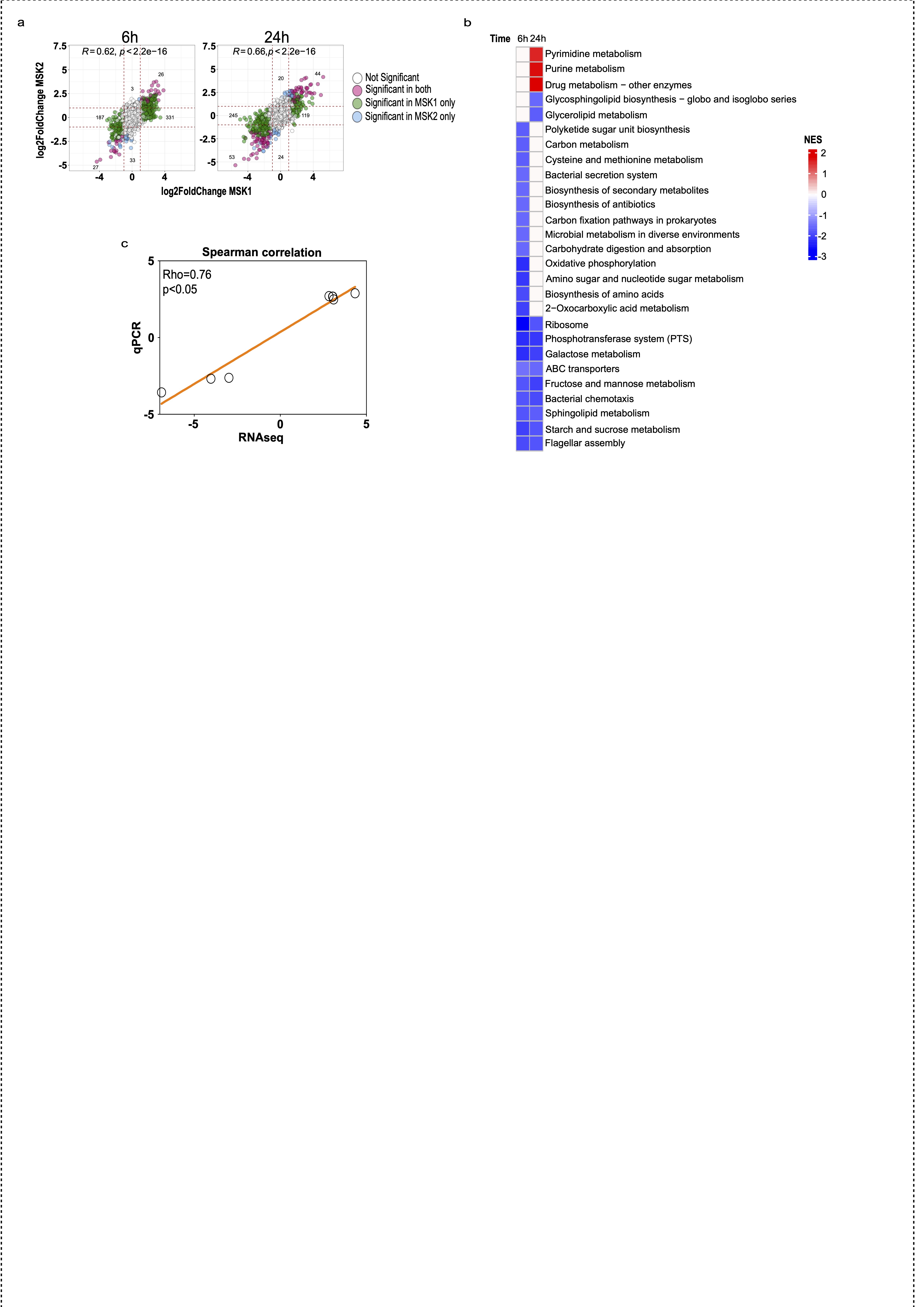

Supplement: Supplemental Material [file KGMI_A_2340486_SM5421.zip › David Supplementary Figures JPEG/Supp 1.jpg]

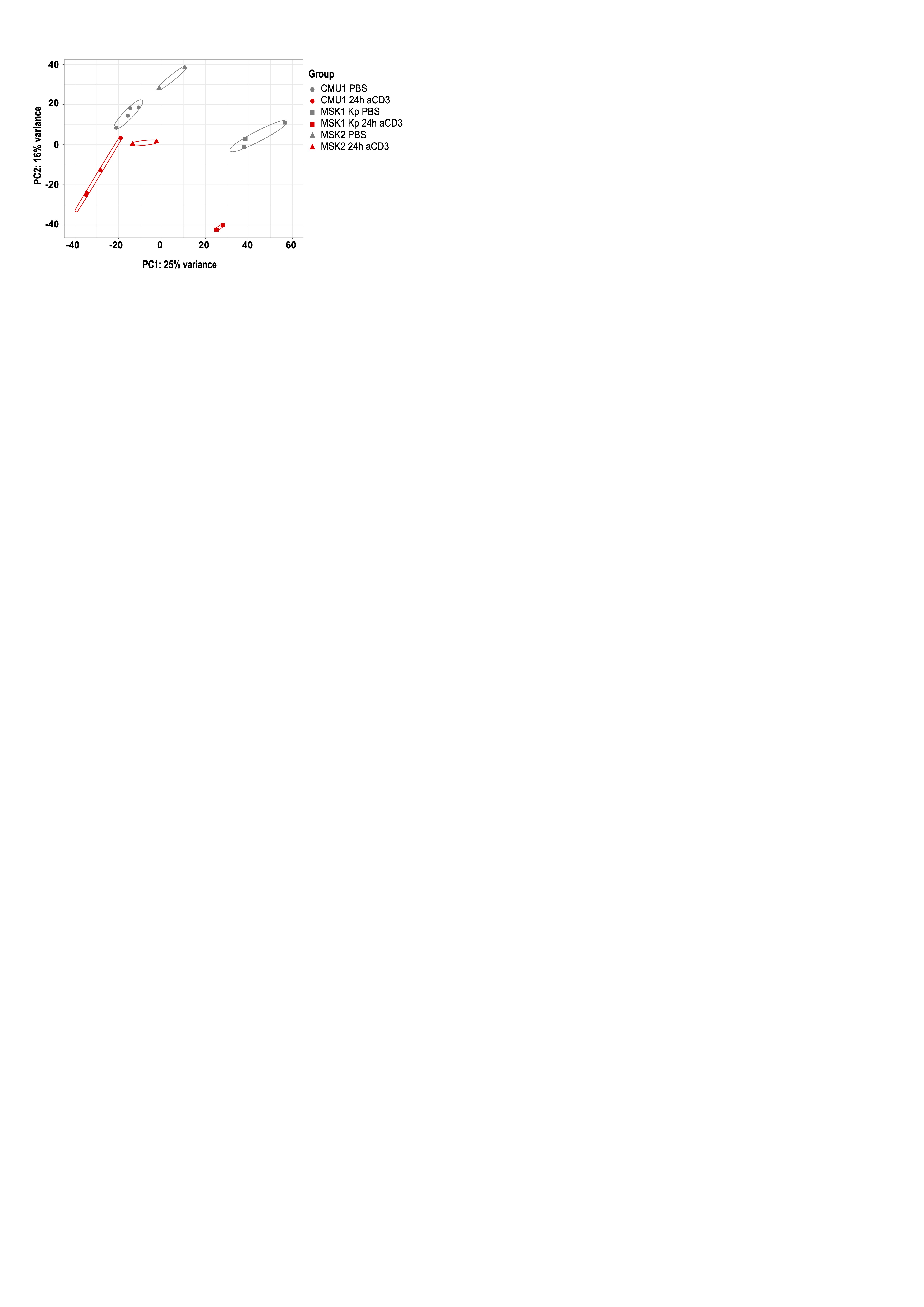

Supplement: Supplemental Material [file KGMI_A_2340486_SM5421.zip › David Supplementary Figures JPEG/Supp 2.jpg]

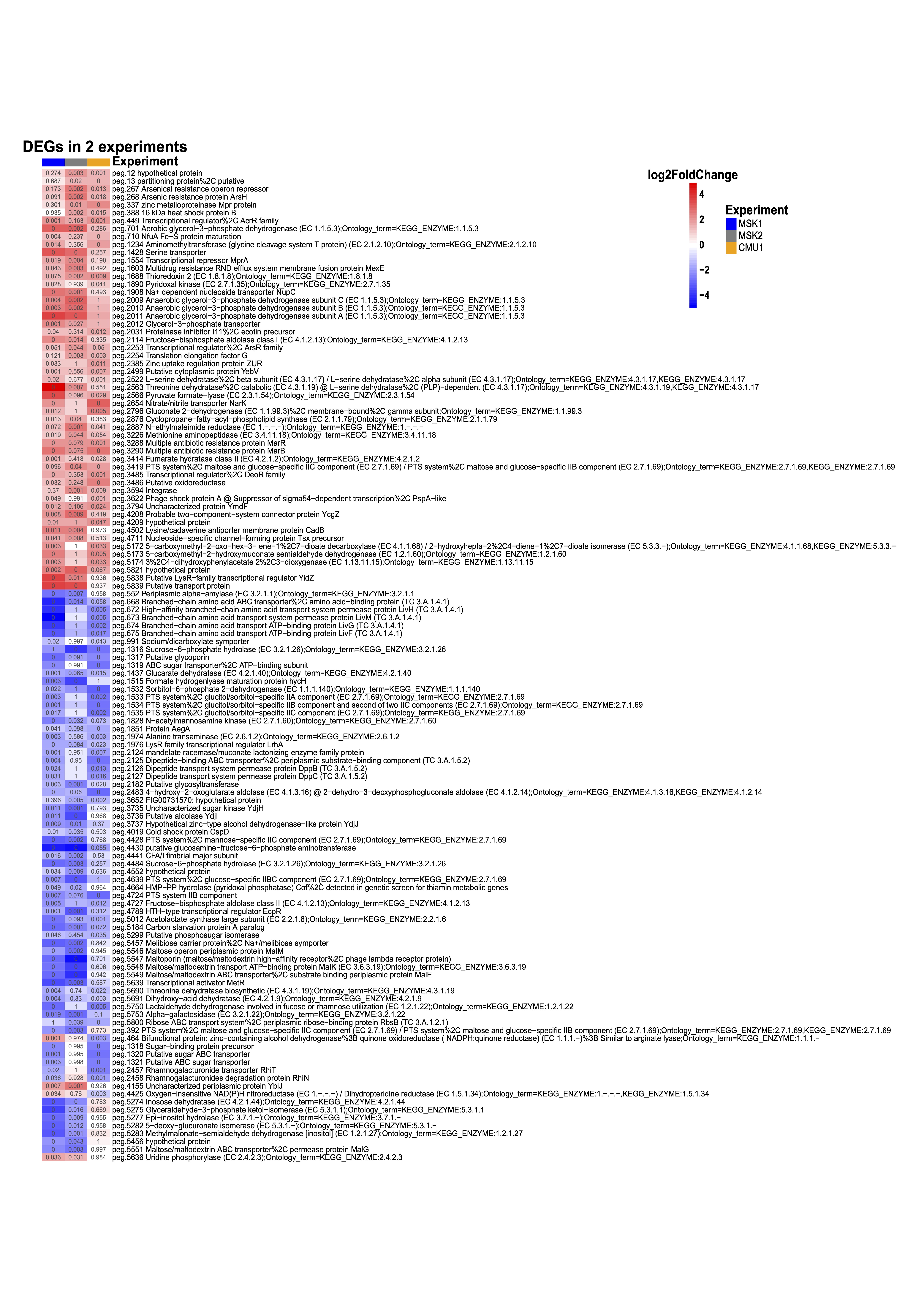

Supplement: Supplemental Material [file KGMI_A_2340486_SM5421.zip › David Supplementary Figures JPEG/Supp 3.jpg]

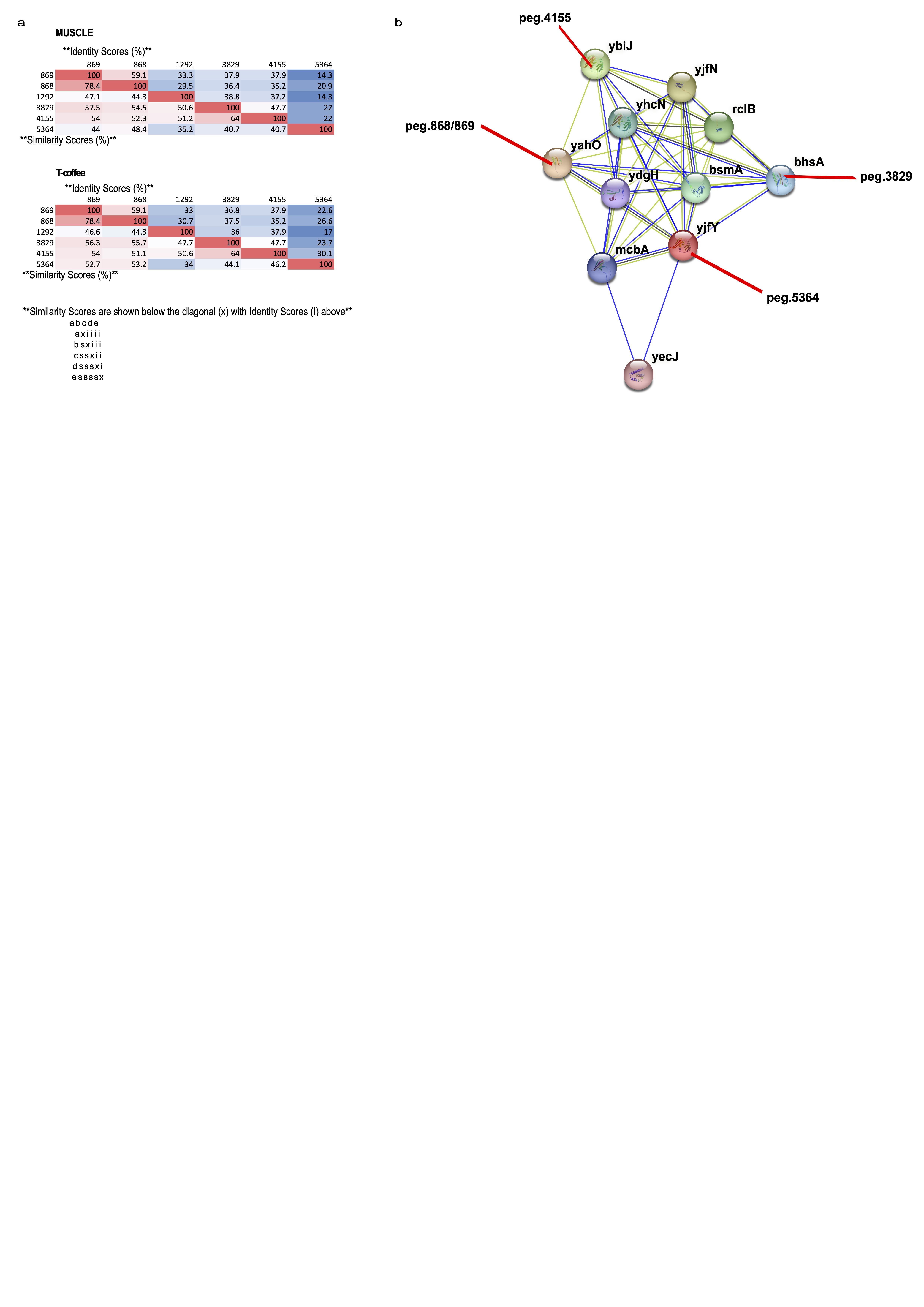

Supplement: Supplemental Material [file KGMI_A_2340486_SM5421.zip › David Supplementary Figures JPEG/Supp 4.jpg]

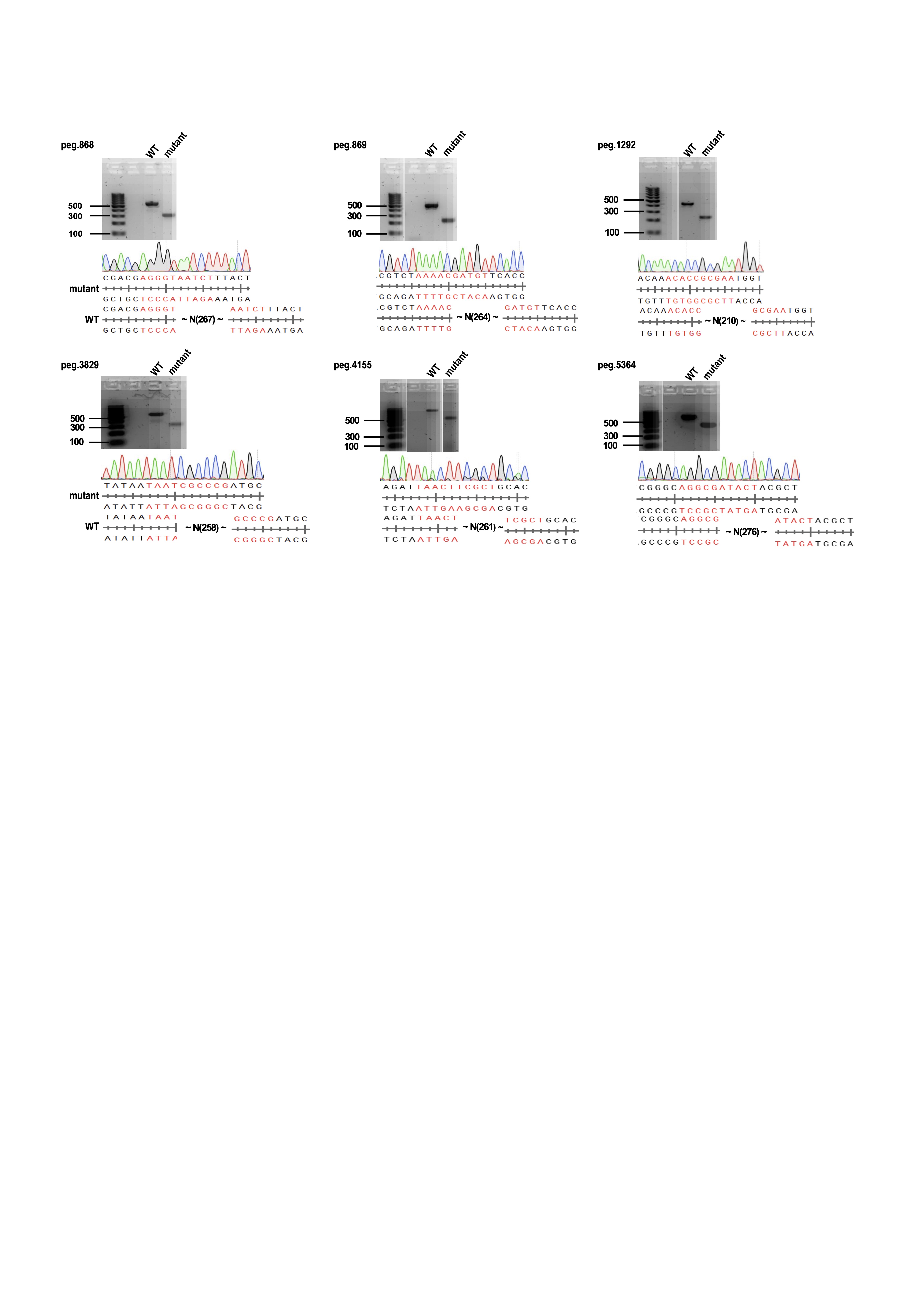

Supplement: Supplemental Material [file KGMI_A_2340486_SM5421.zip › David Supplementary Figures JPEG/Supp 5.jpg]

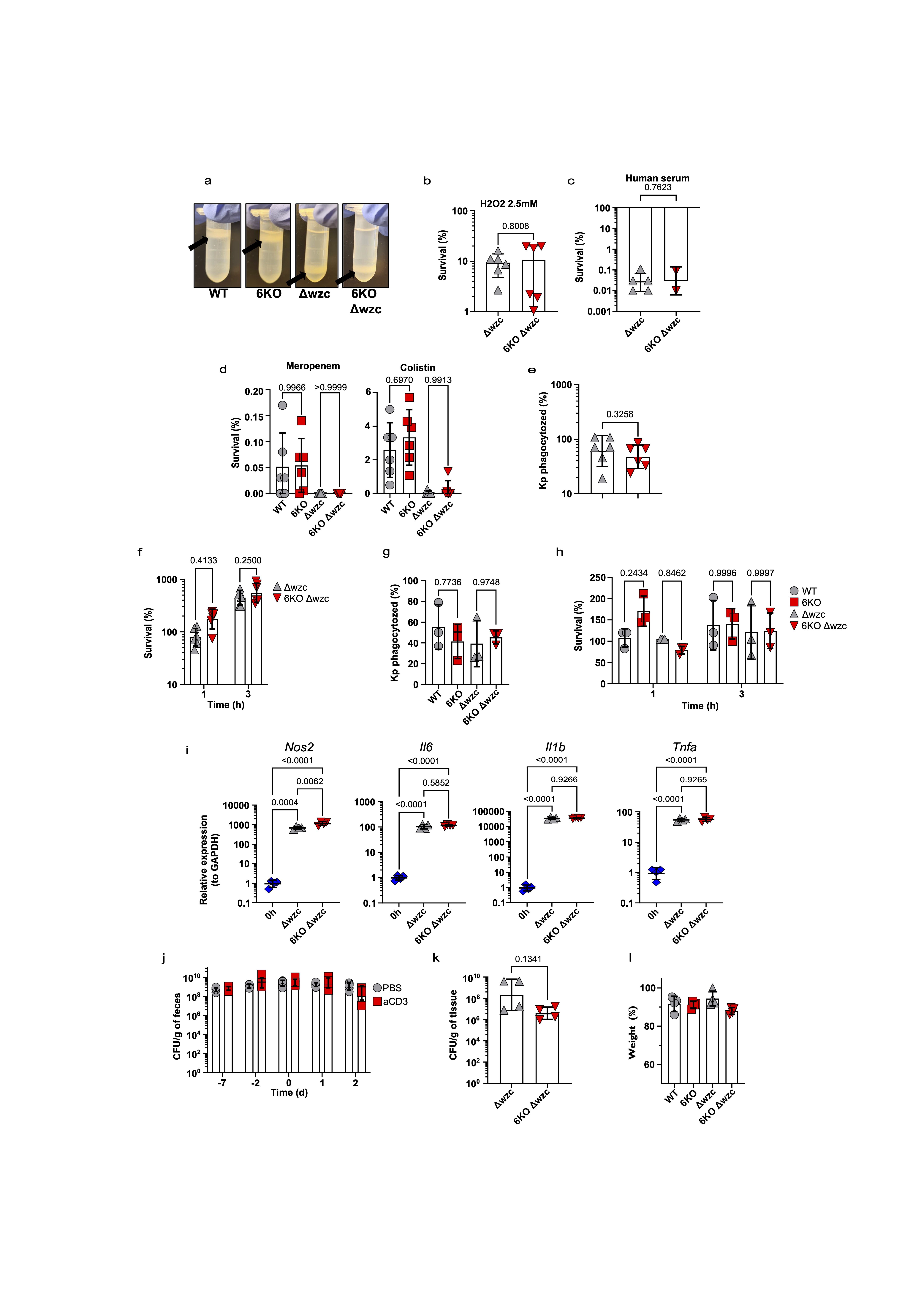

Supplement: Supplemental Material [file KGMI_A_2340486_SM5421.zip › David Supplementary Figures JPEG/Supp 6.jpg]

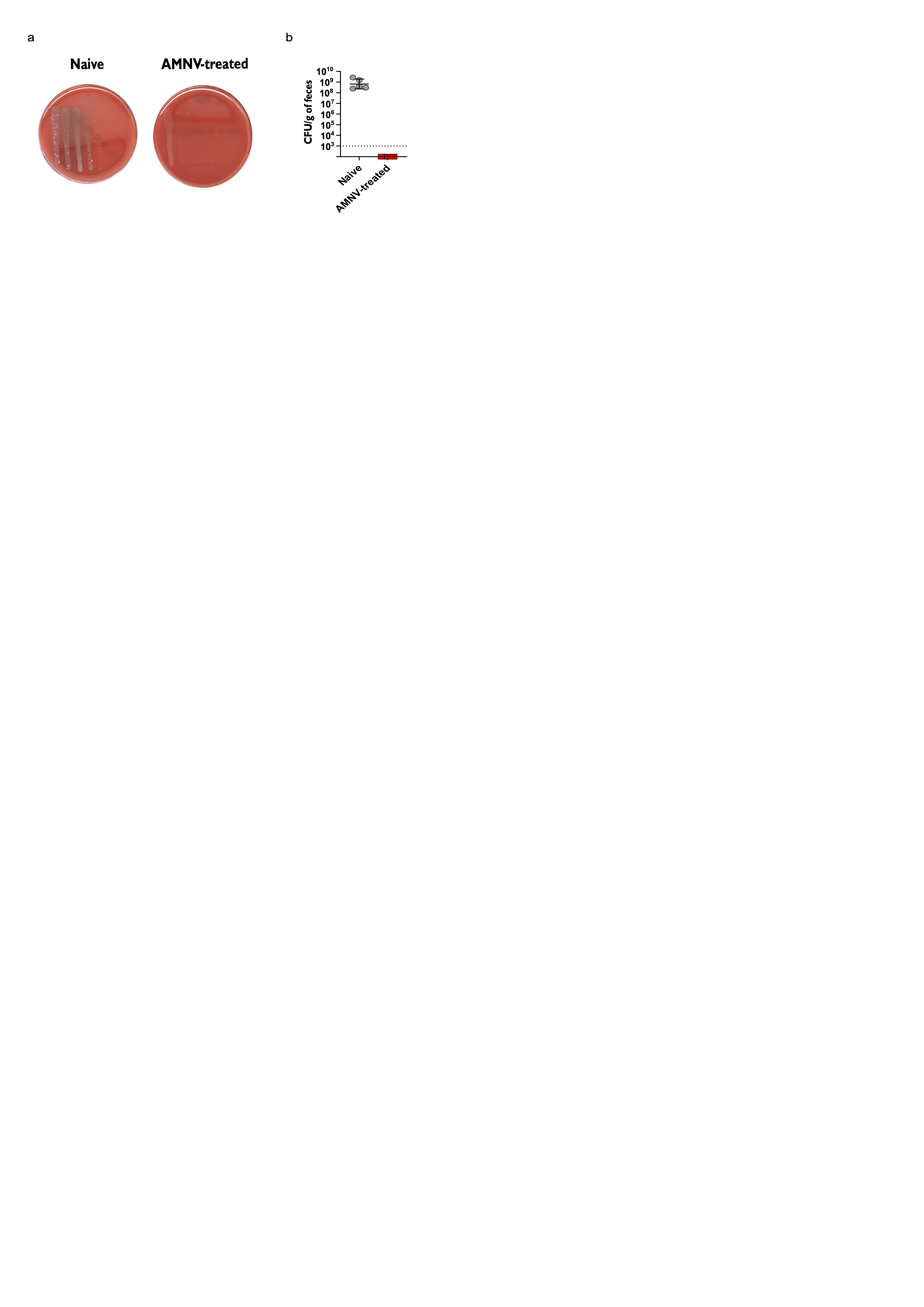

Supplement: Supplemental Material [file KGMI_A_2340486_SM5421.zip › David Supplementary Figures JPEG/Supp 7.jpg]
